# Archaeal *amo*A gene diversity points to distinct biogeography of ammonia-oxidizing *Crenarchaeota* in the ocean

**DOI:** 10.1111/j.1462-2920.2012.02801.x

**Published:** 2013-05

**Authors:** Eva Sintes, Kristin Bergauer, Daniele De Corte, Taichi Yokokawa, Gerhard J Herndl

**Affiliations:** 1 Department of Biological Oceanography, Royal Netherlands Institute for Sea ResearchThe Netherlands; 2 University of Vienna, Department of Marine Biology, Faculty Center of EcologyAustria; 3 Center for Ecological and Evolutionary Studies, University of GroningenThe Netherlands; 4 Center for Marine Environmental Studies, Ehime UniversityMatsuyama, Japan

## Abstract

Mesophilic ammonia-oxidizing *Archaea* (AOA) are abundant in a diverse range of marine environments, including the deep ocean, as revealed by the quantification of the archaeal *amo*A gene encoding the alpha-subunit of the ammonia monooxygenase. Using two different *amo*A primer sets, two distinct ecotypes of marine *Crenarchaeota* Group I (MCGI) were detected in the waters of the tropical Atlantic and the coastal Arctic. The HAC-AOA ecotype (high ammonia concentration AOA) was ≍ 8000 times and 15 times more abundant in the coastal Arctic and the top 300 m layer of the open equatorial Atlantic, respectively, than the LAC-AOA (low ammonia concentration AOA) ecotype. In contrast, the LAC-AOA ecotype dominated the lower meso- and bathypelagic waters of the tropical Atlantic (≍ 50 times more abundant than the HAC-AOA) where ammonia concentrations are well below the detection limit using conventional spectrophotometric or fluorometric methods. Cluster analysis of the sequences from the clone libraries obtained by the two *amo*A primer sets revealed two phylogenetically distinct clusters. Taken together, our results suggest the presence of two ecotypes of archaeal ammonia oxidizers corresponding to the medium (1.24 µM on average in the coastal Arctic) and low ammonia concentration (< 0.01 µM) in the shallow and the deep waters respectively.

## Introduction

One of the major findings in microbial oceanography over the past two decades has been the ubiquitous presence of mesophilic *Archaea* throughout the oceanic water column including the deep ocean (DeLong, 1992; Fuhrman *et al*., 1992; Karner *et al*., 2001). The marine *Crenarchaeota* Group I (MCGI), recently coined *Thaumarchaeota* (Brochier-Armanet *et al*., 2008), is a dynamic component of the prokaryotic community, generally increasing in its contribution to total prokaryotic abundance (PA) with depth (Karner *et al*., 2001; Varela *et al*., 2008).

Early reports on the metabolic activity of marine MCGI showed that they take up amino acids (Ouverney and Fuhrman, 2000; Teira *et al*., 2006) in the mesopelagic and bathypelagic waters. However, MCGI do not simply exhibit a heterotrophic life style as these studies might suggest. In the database generated by the large-scale sequencing effort of Venter and colleagues (2004), the gene for the ammonia monooxygenase subunit A (*amo*A) of apparently archaeal origin was identified, suggesting that at least some members of the MCGI are nitrifiers and hence chemoautotrophs, as indicated earlier by compound-specific lipid analyses (Pearson *et al*., 2001; Wuchter *et al*., 2003). Additionally, the mesophilic crenarchaeal isolate ‘*Candidatus* Nitrosopumilus maritimus’ (Könneke *et al*., 2005) has been shown to incorporate dissolved inorganic carbon (DIC) as carbon source and using ammonia as energy source. This chemoautotrophic life style of MCGI has been subsequently confirmed using various approaches (Ingalls *et al*., 2006; Wuchter *et al*., 2006; Hallam *et al*., 2006b; 2006a; Mincer *et al*., 2007; Hansman *et al*., 2009). Quantitative studies suggest that MCGI are mostly autotrophs (Ingalls *et al*., 2006; Hansman *et al*., 2009). However, evidence of some level of heterotrophy or mixotrophy is also represented in MCGI (Herndl *et al*., 2005; Teira *et al*., 2006; Hallam *et al*., 2006b; Martin-Cuadrado *et al*., 2008).

Studying the distribution of ammonia-oxidizing *Archaea* (AOA) in the main deep water masses of the North Atlantic, a major gradient in AOA abundance was found decreasing from north to south (Agogué*et al*., 2008), coinciding with the generally higher ammonia availability in the northern than in the corresponding (sub)tropical deep water masses (Varela *et al*., 2008). This strong latitudinal gradient was interpreted as an indication that in the lower meso- and bathypelagic waters of the northern North Atlantic, ammonia-oxidizing MCGI prevail, while towards the equator, MCGI are primarily depending on energy sources other than ammonia (Agogué*et al*., 2008).

In the deep waters of the North Pacific Gyre, however, crenarchaeal *amo*A gene abundance was reported to remain rather stable down to 1000 m depth (Mincer *et al*., 2007; Beman *et al*., 2008; Church *et al*., 2010). This apparent discrepancy between the deep water MCGI in the lower latitudes of the Atlantic and Pacific in harbouring the *amo*A gene can be caused by (i) biogeographic differences in MCGI in their ability to oxidize ammonia, and (ii) methodological differences due to, e.g. different primer sets used. This latter explanation seems to be the more plausible as suggested by the finding of three mismatches between the reverse primer used for q-PCR analysis of archaeal *amo*A in the North Atlantic study (Agogué*et al*., 2008) and the genomic scaffold sequence obtained from MCGI at 4000 m depth in the deep Pacific (Konstantinidis *et al*., 2009).

The existence of different AOA clusters has been pointed out previously (Francis *et al*., 2005), subsequently suggested to represent vertically segregated groups (Hallam *et al*., 2006a). Although the relationships of these two groups with ammonia oxidation rates were explored in the Gulf of California (Beman *et al*., 2008), only the surface water AOA cluster was suggested to be actively involved in ammonia oxidation as indicated by the correlation between surface water AOA abundance and the ammonia oxidation rates. Thus, the ecological implication of the occurrence of different groups of AOA and particularly the role and biogeography of the ‘deep’ AOA group remain enigmatic.

We hypothesized that AOA exhibit a distribution pattern with clusters adapted to lower and higher ammonia supply rates. Higher ammonia supply rates are generally expected in subsurface waters (∼ 100 m depth) and upper mesopelagic waters (150–500 m depth) (Brzezinski, 1988; Murray *et al*., 1989; Sambrotto, 2001; Woodward and Rees, 2001), while low ammonia supply rates and ammonia concentrations well below the detection limit of conventional fluorometric methods (< 10 nM) are found in lower meso- and bathypelagic waters (500–4000 m depth) (Cline and Richards, 1972; Brzezinski, 1988; Varela *et al*., 2008). In polar deep waters, generally higher ammonia supply rates are found than in the deep waters near the equator (Woodward and Rees, 2001; Varela *et al*., 2008). Hence, we hypothesized a biogeographic and depth-related distribution pattern of AOA clusters corresponding to the different supply rates of ammonia.

This hypothesis was tested using two primer sets to determine archaeal *amo*A gene abundance by q-PCR and establishing clone libraries: the primer set developed from AOA collected in the North Sea (Wuchter *et al*., 2006), targeting the *amo*A gene of the ammonia monooxygenase putatively adapted to higher ambient ammonia concentrations. The second primer set, using the reverse primer based on the sequence of the genomic scaffold obtained from MCGI collected in the subtropical Pacific Gyre (Konstantinidis *et al*., 2009), target presumably the *amo*A gene encoding the ammonia monooxygenase for low ambient ammonia concentrations. These two primer sets, coined thereafter ‘low-’ and ‘high-ammonia concentration primer set’, were used to quantify *amo*A gene abundance in waters of contrasting trophic status and over a large depth range (Fig. S1). The Romanche Fracture Zone of the tropical Atlantic is an oligotrophic open ocean site with a water column extending to 7500 m depth and the coastal Arctic as a meso- to eutrophic site with comparatively high (on average 1.24 µM) ammonia concentrations.

## Results

### Prokaryotic abundance and activity in the two oceanic regions

Prokaryotic abundance (PA) and heterotrophic activity (PHA), measured as leucine incorporation, both decreased exponentially with depth at both study sites (Fig. S2A and B). PA varied between 2.3 and 14.4 × 10^5^ cells ml^−1^ (300 to 1.5 m depth) in the Arctic (Fig. S2A) and between 0.1 and 2.7 × 10^5^ cells ml^−1^ (7000 to 100 m depth) at the Atlantic stations (Fig. S2B). PHA followed a similar trend as PA; however, it was generally substantially higher in the coastal waters of the Arctic site (2.52–104 pmol Leu l^−1^ h^−1^) than in the meso- to abyssopelagic waters of the Romanche Fracture Zone (0.002–5.88 pmol Leu l^−1^ h^−1^) (Fig. S2A and B).

Dark DIC fixation rates were high in the coastal Arctic, ranging from 37 to 458 µmol C m^−3^ day^−1^ and decreasing exponentially with depth. The dark DIC fixation rates in the Atlantic ranged between 0.01 and 14.09 µmol C m^−3^ day^−1^, with highest values in the subsurface layer and the oxygen minimum zone (Fig. S2C).

### Specificity of the two primer sets

The *amo*A gene abundance from clones belonging to the tentative HAC (‘high-ammonia concentration’) cluster detected using the LAC (‘low-ammonia concentration’) primer set varied between 0.00% and 6.6% of the gene abundance determined with the HAC primer set for the same clones, with median values ranging from 0.04% to 1.2% for high and low gene abundance respectively (Table S1). The gene abundance of the clones associated to the LAC cluster detected with the HAC primer set ranged between 0.01–8.98% (median 0.64–1.59%) of their abundance determined with the LAC primer set (Table S1).

Mixtures of clones belonging to the two clusters in different proportions, to mimic a AOA community with HAC- and LAC-*amo*A, showed no significant differences between the proportion of HAC- or LAC- clones added to the mixture and the proportions obtained with the respective primer set (paired *t*-test, *P* = 1.0). The added and the measured proportions were linearly correlated (slope 0.98 ± 0.02, *r*^2^ = 0.994, *P* < 0.001) (Fig. S3).

### Abundance and distribution of archaeal *amo*A genes detected with the two primer sets

**Fig. 1:**
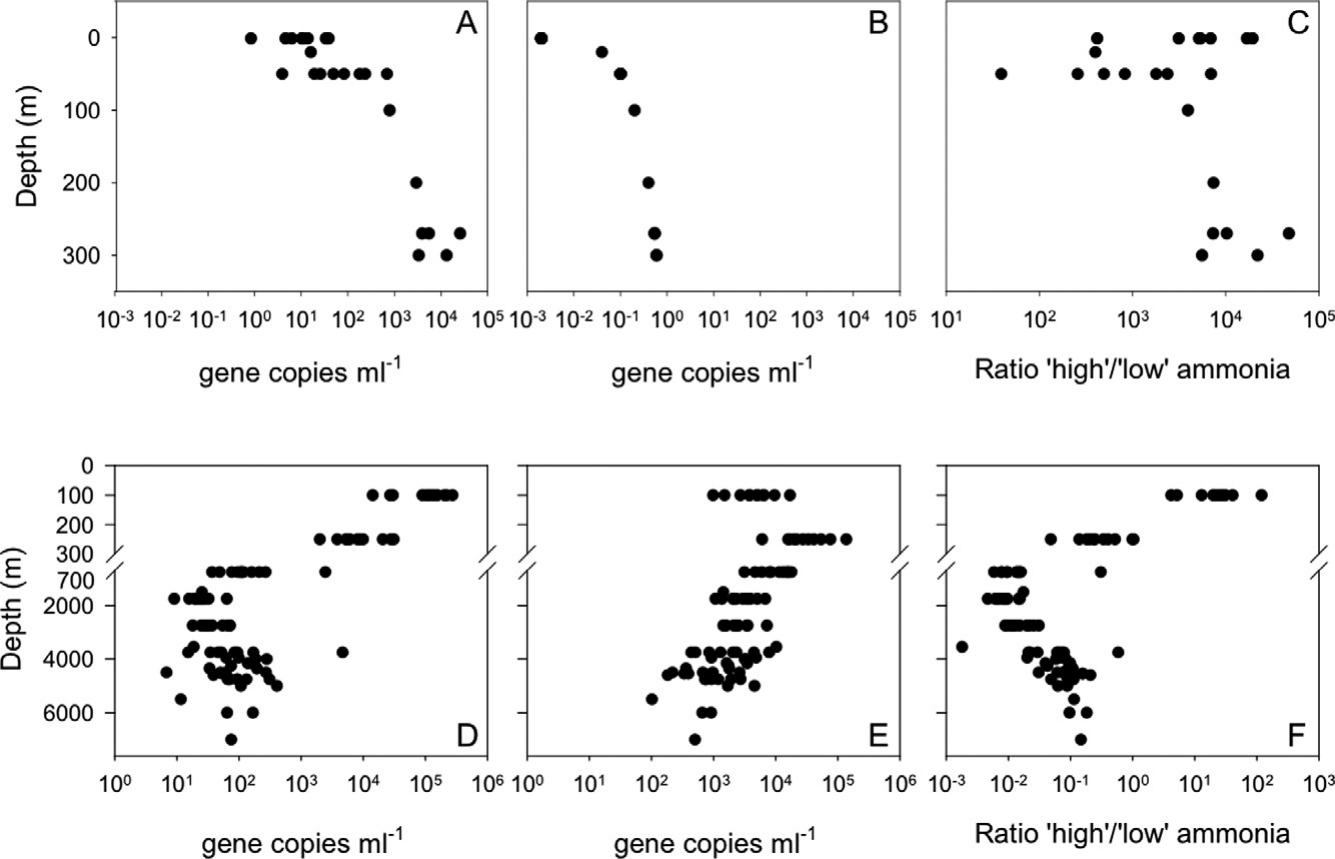
Archaeal *amo*A gene abundance in the Arctic: (A) obtained with the ‘high-ammonia concentration’ primer set; (B) obtained with the ‘low-ammonia concentration’ primer set; (C) ratio between archaeal *amo*A gene abundance obtained with the ‘high’ versus ‘low ammonia concentration’ primer. Depth profiles of archaeal *amo*A gene abundance in the Atlantic: (D) obtained with the ‘high-ammonia concentration’ primer set; (E) obtained with the ‘low-ammonia concentration’ primer set; (F) ratio between archaeal *amo*A gene abundance obtained with the ‘high’ versus ‘low ammonia concentration’ primer.

In the Arctic, archaeal *amo*A gene abundance increased by 2 and 3 orders of magnitude with the LAC and HAC primer set, respectively, from 1.5 to 300 m depth (Fig. 1A and B, Table S2). The ratio of *amo*A gene abundance detected with the HAC and LAC primer set varied between ≍ 100–5 × 10^4^ in the Arctic, with lowest ratios at 50 m depth (Fig. 1C, Table S2).

In the Atlantic, archaeal *amo*A gene abundance obtained with the HAC primer set was highest in the subsurface layers with an average *amo*A gene abundance of 1.25 × 10^5^ ml^−1^ decreasing to an average of 120 ml^−1^ in the abyssopelagic layer (Fig. 1D, Table S2). The archaeal *amo*A gene abundance obtained with the LAC primer set was 5.7 × 10^3^ ml^−1^ in the surface layer, increasing towards the oxygen minimum layer (4.1 × 10^4^ ml^−1^), and decreasing towards the abyssopelagic waters to 1.6 × 10^3^ ml^−1^ (Fig. 1E, Table S3). Taken together, archaeal *amo*A gene abundance determined with the HAC *amo*A primer set was higher than that obtained with the LAC *amo*A primer set in the surface waters down to approximately 100 m depth (Fig. 1D and E, Fig. S4A and B). In contrast, below 250 m depth, archaeal *amo*A gene abundance obtained with the LAC primer set was higher than with the HAC primer set (Fig. 1D and E, Fig. S4A and B). The ratio between the archaeal *amo*A gene abundance obtained with the HAC versus LAC primer set was highest at 100 m depth with an average of 30, decreasing exponentially with depth averaging 0.01 at 1750 m depth, and increasing again to a mean ratio of 0.1 in the abyssopelagic layer (Figs 1F and S4C).

**Fig. 2:**
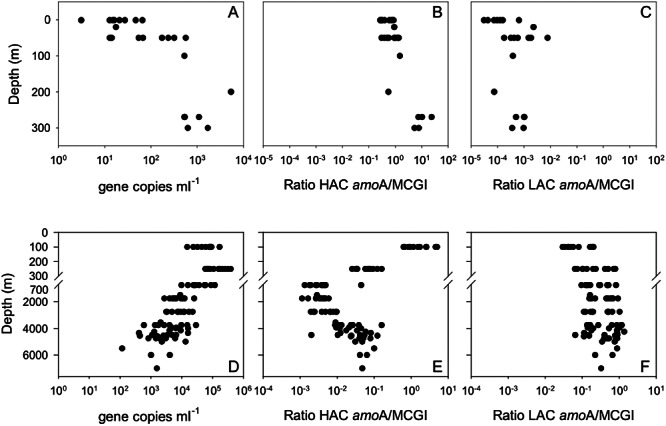
Depth profiles of (A) abundance of Marine *Crenarcheaota* Group I (MCGI) genes in the coastal Arctic and the ratio archaeal *amo*A gene abundance versus MCGI 16S rDNA gene abundance obtained with (B) the ‘high-ammonia concentration’ primer set and (C) with the ‘low-ammonia concentration’ primer set. Marine *Crenarchaeota* 16S rDNA gene abundance in the Atlantic (D), and ratio ‘high-ammonia concentration’*amo*A gene (E) and ratio ‘low-ammonia concentration’*amo*A gene (F) versus MCGI 16S rDNA.

MCGI 16S rRNA gene abundance exhibited a similar trend with depth as archaeal *amo*A gene abundance at both the Arctic (Fig. 2A, Table S2) and the tropical Atlantic site (Fig. 2D, Table S3, Fig. S5A). The ratio of HAC archaeal *amo*A to MCGI 16S rRNA gene abundance ranged between 1 and 10 in the Arctic (Table S2, Fig. 2B), while the ratio between the LAC archaeal *amo*A and MCGI 16S rRNA gene abundance was very low ranging between 10^−4^ and 10^−2^ (Table S2, Fig. 2C).

In the Atlantic, the ratio of HAC to MCGI 16S rRNA gene abundance was higher than 1 down to 250 m depth (Table S3, Fig. 2E). From 750 to 2750 m depth, the ratio of HAC archaeal *amo*A to MCGI 16S rRNA gene abundance was very low (on average 5 × 10^−3^), slightly increasing again at depths below 2750 m (Fig. 2E, Table S3, Fig. S5B). In the Atlantic, the ratio between the LAC archaeal *amo*A and MCGI 16S rRNA gene abundance was generally lower than 1, reaching unity in the deep waters (below 2750 m) in the central part of the Romanche Fracture Zone (Fig. 2F, Table S3, Fig. S5C). Thus, the spatial distribution of the ratio between ‘total’ archaeal *amo*A and MCGI 16S rRNA gene abundance resulted in values ranging from 0.6 to 5.0 in surface waters and ratios close to 1 in deep waters of the central part of the Romanche Fracture Zone (Fig. S6).

### Phylogeny of *amo*A clones

There was no consistent trend with depth (two-way anova, *P* > 0.7) in the number of OTUs obtained by cloning with the HAC and the LAC primers as indicated by the rarefaction curves shown in Fig. S7.

**Fig. 3:**
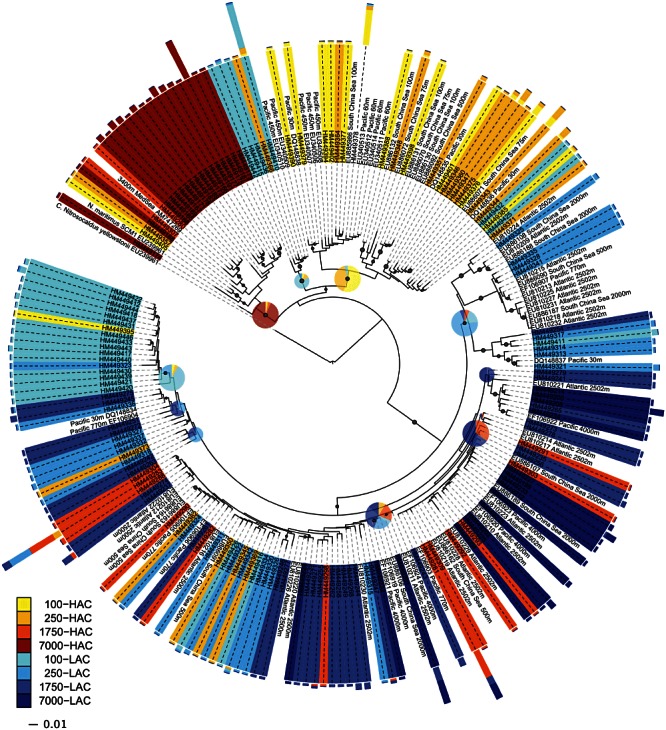
Phylogenetic tree of archaeal *amo*A sequences recovered from the subtropical Atlantic with the ‘high ammonia concentration’ (yellow–red tones) and the ‘low ammonia concentration’ (blue tones) primer sets, NCBI database sequences (black). Light to dark tones: 100–7000 m depth. One representative of sequence group ≥ 99% identical is shown; the bar shows the number of clones represented by a sequence. Proportion of clones represented by the different clusters is indicated by the pie charts at the branch internal nodes. Bootstrap values (> 50%) are indicated by the grey circle at branch point.

The clones obtained with both primer sets clustered with archaeal *amo*A from *Nitrosopumilus maritimus* and other environmental clones deposited at NCBI (Fig. 3), but not with bacterial *amo*A. Clones obtained with the two primer sets, however, rarely clustered together. Differences between the AOA clone libraries obtained with the two primer sets were tested using UniFrac. With the HAC primer set, the obtained clones were significantly different from those obtained with the LAC primer in the total number of clones (*P* < 0.01, UniFrac significance analysis using Bonferroni correction). The clones obtained with the HAC and the LAC primers were significantly different at each of the depth layers (*P* < 0.001, UniFrac significance analysis; Fig. S8), with the exception of the clones from 100 m and 250 m depth obtained with the HAC primer set (*P* = 0.66). *Nitrosopumilus maritimus* clustered with some clones from 100 and 7000 m depth obtained with the HAC primer (Fig. 3). Clones obtained with the HAC primer set clustered with NCBI sequences from surface waters and oxygen minimum zones, while the clones obtained with the LAC primer set clustered with sequences from oxygen minimum zones and deep waters from several regions of the ocean (Fig. 3).

## Discussion

### Specificity of the two primer sets to target different AOA clusters

The two sets of primers used in this study target two different clusters of AOA with high specificity (Table S1). The percentage of amplification of members of one cluster with the differing primer set with a median < 1.59% at any gene abundance is consequently not significantly affecting the total *amo*A gene abundance. Nevertheless, in some environments the AOA abundance obtained with one primer set might be lower than 1% of the abundance obtained with the other primer set, e.g. at 1750 m in the Atlantic stations (Table S3) or in the coastal Arctic (Table S2). Thus, it might be that one of the clusters is absent. However, this 1% detection limit is a sort of worst-case scenario when the DNA source is one single clone and thus, unspecific binding of the primer might be overestimated. In mixed DNA sources, similar to natural communities, the proportion of the two different clusters added and measured are related at least down to 0.2% (Fig. S3).

The apparently contrasting result obtained by cloning with the LAC primer set at the surface and the HAC primer set at 1750 m depth, where the archaeal *amo*A obtained with these primer sets mostly clusters together with the differing cluster (Fig. 3), can be explained by the very low abundance of the HAC-AOA at 1750 m as compared with the LAC-AOA (HAC/LAC 0.005–0.016) and the high abundance of HAC-AOA at 100 m depth. The cloning effort was continued until enough DNA was amplified and sufficient colonies grew, but under severe dominance of either the HAC- or the LAC-AOA group, the unspecific binding of the primer to the available DNA, although low, might have been significant.

### Biogeography of archaeal ammonia oxidizers obtained with the two primer sets

The HAC primer set recovered most of the sequences that can be obtained also with other *amo*A primer sets (Francis *et al*., 2005; Beman *et al*., 2008), with the exception of some deep water sequences. However, the HAC primer set does not amplify *amo*A from the LAC-AOA, which were recovered in this study by the LAC primer set developed from the sequence information from bathypelagic MCGI (Konstantinidis *et al*., 2009). Beman and colleagues (2008) described two vertically segregated groups of AOA in the Gulf of California, which were targeted by different specific primers. Thus, it appears that using only one primer set does not recover all the diversity of the crenarchaeal ammonia oxidizers.

Corresponding to the higher recovery of surface AOA with the HAC primer set in our study, the ratio of HAC versus LAC archaeal *amo*A was high (range 4–120) at 100 m depth in the Atlantic, while in the deep layers, this ratio was always lower than 1 (Fig. 1F and Fig. S4C). In the Arctic, AOA were dominated by the HAC *amo*A cluster at all depths, being 2–5 orders of magnitude more abundant than the LAC *amo*A gene (Fig. 1C, Table S2).

In the Atlantic, the ratio total (i.e. HAC + LAC) *amo*A : MCGI 16S rRNA gene abundance reached values close to unity towards the centre of the Romanche Fracture Zone (Fig. S6), in contrast to an earlier study (Agogué*et al*., 2008) targeting only the HAC-AOA but in agreement with other studies (Mincer *et al*., 2007; Beman *et al*., 2008; Church *et al*., 2010). Thus, the results obtained in the present study support the notion that MCGI have the potential to oxidize ammonia throughout the water column even in bathypelagic waters. This finding is in contrast to that reported by Agogué and colleagues (2008) for the temperate and subtropical bathypelagic Atlantic waters. However, Agogué and colleagues (2008) used the primer set that targets only the HAC-AOA, thus underestimating the total abundance of AOA, particularly in the meso- and bathypelagic waters of the temperate to equatorial Atlantic. Consequently, the gradients reported by Agogué and colleagues (2008) refer to the HAC-AOA and not to the total archaeal ammonia oxidizers community. Whether ammonia is actually used as an energy source in bathypelagic AOA remains to be shown however, since the ammonia concentrations in the bathypelagic waters are below 10 nM concentrations (the detection limit of the fluorometric method to determine NH_3_).

Archaeal *amo*A : MCGI 16S rRNA gene abundance ratios higher than 1 are usually found in surface waters (Wuchter *et al*., 2006; Agogué*et al*., 2008; Church *et al*., 2010; Christman *et al*., 2011). The thus far fully sequenced marine archaeal species, *N. maritimus, C. symbiosum* and *Candidatus* Nitrosoarchaeum limnia, have one *amo*A gene per genome (Hallam *et al*., 2006b; Walker *et al*., 2010; Blainey *et al*., 2011). A possible explanation for archaeal *amo*A : MCGI 16S rRNA gene abundance ratios higher than unity could be due to the primer coverage of MCGI in surface waters (De Corte *et al*., 2009). As reported for deep sea marine sediments (Teske and Sorensen, 2008) some lineages of *Archaea* might not be efficiently amplified by PCR due to the presence of mismatches in the primer sequences. An additional explanation might be the presence of the psL12 group of *Crenarchaeota* in the (sub)tropical Atlantic as previously reported (Agogué*et al*., 2008), which may also contain *amo*A and was not quantified in the present study.

The clones obtained with the LAC and the HAC primer sets represent phylogenetically distinct clusters (Fig. 3, Fig. S8), resulting in a distinct distribution pattern of AOA, indicating stratification of AOA in the water column (Figs 3 and S8) and a biogeographic distribution pattern (Figs 1 and 2, and Figs S4 and S5). The different AOA clusters obtained from waters of the California Current (Beman *et al*., 2010; Santoro *et al*., 2010) further support the notion of stratification of AOA in different oceans. Also, the available archaeal *amo*A sequences in the NCBI database targeted with both sets of primers indicate the dominance of HAC-AOA in most environments with the exception of the deep sea (Table S4). Since the deep ocean is the largest habitat in the biosphere (Arístegui *et al*., 2009), the LAC-AOA might be numerically important, albeit still uncultured.

### Niche separation of the two AOA clusters according to ammonia supply rates

There is indication that ammonia concentration is an important factor determining the rates of nitrification and the abundance of archaeal and bacterial ammonia oxidizers in the Arctic (Christman *et al*., 2011). We found a predominance of the LAC-AOA in deeper layers, where ammonia is below detectable levels using routine spectrophotometric and –fluorescence methods (Brzezinski, 1988; Varela *et al*., 2008). In contrast, a predominance of HAC-AOA was detected in surface waters and upper mesopelagic layers of the Atlantic where ammonia is present at concentrations of 20–100 nM (Clark *et al*., 2008) or even higher in the boreal regions of the Atlantic (Woodward and Rees, 2001; Varela *et al*., 2008) and where nitrification rates are readily measurable (Santoro *et al*., 2010; Christman *et al*., 2011). These results are also in agreement with the predominance of the HAC-AOA in the Arctic where ammonia concentrations are higher than in the low latitude Atlantic, ranging between 0.09 and 2.58 µM (Table S2, Fig. S9). Moreover, our conclusion that there might be two clusters of AOA with a distribution depending on the environmental ammonia concentration is further supported by the negative correlation between ammonium concentration and the ratio LAC- versus HAC-*amoA* (*r* = −0.38, *P* < 0.0005 for the full data set), and between nitrite concentrations, the product of the ammonia oxidation, and the ratio LAC- versus HAC-*amo*A (*r* = −0.72 and *r* = −0.82, *P* < 0.0001, for the Arctic and Atlantic samples, respectively). LAC- and HAC-AOA abundance positively correlated with nitrite concentrations in the Arctic (*r* = 0.93 and *r* = 0.69 respectively), whereas in the Atlantic only the HAC-AOA abundance positively correlated with nitrite concentrations (*r* = 0.62). Consequently, the negative relationship between the ratio LAC/HAC and the concentration of nitrite supports the notion of a dominance of HAC in environments with higher ammonia supply rates.

Different mechanisms might determine the relationship between the two AOA clusters and nutrient concentrations, such as different affinity for ammonia, the presence of different ammonia permeases or different concentrating mechanisms. Thus, further research is needed to clarify the nature of the mediators of ammonia oxidation in the oceans, and to clarify the role of the different subunits of the *amo* protein in the ammonia oxidation.

In summary, we have shown that ammonia-oxidizing mesophilic marine *Crenarchaeota* do apparently exhibit a distinct biogeographic distribution pattern in the North Atlantic with distinct clusters governed by the prevailing ammonia supply rates.

## Experimental procedures

### Sampling and study areas

Sampling was conducted at two different sites. During the Archimedes-III cruise with R/V *Pelagia*, water was collected in the tropical Atlantic between 20 December 2007 and 16 January 2008, and in the coastal Arctic during the PACCA-07 campaign in Aug 2007. Water samples during the Archimedes-III cruise were taken at 17 stations (Fig. S1A) with 10 l Niskin bottles mounted in a frame holding also sensors for conductivity-temperature-depth (CTD), salinity, oxygen, fluorescence and optical backscattering. Water samples were collected from the lower euphotic layer (100 m depth), oxygen minimum zone (250 and 750 m depth), and bathy- and abyssopelagic depths (1750–7000 m). Water samples during the PACCA campaign were taken at eight stations (Fig. S1B) located along a transect through the Kongsfjorden Bay at Svalbard, Spitsbergen, Norway, with 10 l Niskin bottles attached to a CTD frame also holding sensors for salinity, chlorophyll fluorescence and optical backscattering. Samples were taken at the surface (1–2 m depth), at ≍ 50 m depth and about 5 m above bottom (between 50 and 300 m depth).

### Inorganic nutrient concentrations

The concentrations of dissolved inorganic nutrients (NH_4_^+^, NO_3_^-^, NO_2_^-^, PO_4_^3−^) were determined after filtering the samples through 0.2 µm filters (Acrodisc, Gelman Science) in a TRAACS 800 autoanalyser system as described elsewhere (Reinthaler *et al*., 2008). A stock solution of 1.109 mM of ammonium chloride was used to prepare the standard curve for NH_4_^+^ concentration measurements, with nine dilutions between 0 and 4.5 µM. The correlation coefficients for the standard curves obtained with the indophenol blue method were ≥ 0.9999, the standard deviation was 0.015 and 0.020 µM within and between runs respectively. The NH_4_^+^ concentration at the Atlantic sites was analysed fluorometrically following the protocol A (80 ml sample volume) of Holmes and colleagues (1999). Standard curves were prepared from a stock solution of 1.109 mM ammonium chloride with 5 concentrations ranging between 0 and 0.33 µM. Correlation coefficients were ≥ 0.997. The indophenol blue and the fluorometric method used here for ammonia measurements have been shown to give very similar results (Holmes *et al*., 1999).

### Abundance and activity of the prokaryotic community

Two-millilitre samples were fixed with glutaraldehyde (0.5% final concentration), shock-frozen in liquid N_2_ and kept at −80°C until analysis. To enumerate prokaryotes by flow cytometry, samples were thawed to room temperature and 0.5 ml subsamples stained with SYBR Green I in the dark for 10 min and subsequently, 1 × 10^5^ ml^−1^ of 1 µm fluorescent polystyrene beads (Molecular Probes, Invitrogen) was added to each sample as internal standard. The prokaryotes were enumerated on a FACScalibur flow cytometer (Becton Dickinson) based on their signature in a plot of green fluorescence *versus* side scatter.

To estimate the heterotrophic prokaryotic activity, ^3^H-leucine incorporation, referred herein as prokaryotic heterotrophic production (PHP), was measured in duplicate 5 ml samples and one formaldehyde-killed blank for coastal Arctic waters and 10–40 ml triplicate samples and blanks for the open Atlantic waters. ^3^H-leucine (20 and 5 nM final concentration for the Arctic and the Atlantic, respectively, Amersham, specific activity 160 Ci mmol^−1^) was added to the samples and blanks and incubated in the dark at *in situ* temperature for 4–24 h (depending on the expected abundance and activity of the microbial community). Subsequently, the samples were fixed with formaldehyde (2% final concentration), filtered onto 0.2 µm polycarbonate filters (Millipore), supported by 0.45 µm HAWP (Millipore) filters, and rinsed three times with 10 ml of 5% ice-cold TCA. Thereafter, the filters were transferred into scintillation vials and dried at room temperature. Subsequently, 8 ml of scintillation cocktail (Packard Filter Count) was added to each vial and counted in a TriCarb 2000 (Perkin Elmer) liquid scintillation counter after 18 h. The mean disintegrations per minute (DPM) were corrected for the corresponding blanks and the leucine incorporation rate calculated.

^14^C-bicarbonate fixation in the dark was used to determine the uptake of inorganic carbon by the prokaryotic community as described previously (Herndl *et al*., 2005). Briefly, 40 ml water samples (in duplicate and one formaldehyde-fixed blank for Arctic and in triplicate samples and blanks for the Atlantic waters) were spiked with ^14^C-bicarbonate (10 and 100 µCi in Arctic and Atlantic respectively; SA, 54.0 mCi mmol^−1^; Amersham) and incubated in the dark at *in situ* temperature for 48–72 h. Subsequently, the samples were fixed with formaldehyde (2% final concentration), filtered onto 0.22 µm filters (Millipore, polycarbonate), and rinsed three times with 10 ml of ultra-filtered seawater (30 kDa molecular mass cut-off). Thereafter, the filters were exposed to a fume of concentrated HCl for 12 h, and subsequently placed in scintillation vials and stored in the dark at −20°C until counted in the scintillation counter. The resulting DPM of the samples were corrected for the DPM of the blank and converted into DIC fixation rates.

### DNA extraction

Ten litres and 1.5 l of seawater for the Atlantic and the coastal Arctic samples, respectively, were filtered through 0.22 µm Sterivex filter GP unit (Millipore). Subsequently, 1.8 ml of lysis buffer (40 mM EDTA, 50 mM Tris-HCl, 0.75 M sucrose) was added to the filters and stored at −80°C until processed in the home laboratory. The extraction was performed using Ultraclean Mega soil DNA isolation kit (Mobio), and the DNA extract was further concentrated using Centricon units (Millipore).

### Preparation of the q-PCR standards

The standards for the 16S rDNA of Marine *Crenarchaeota* Group I (MCGI) and the archaeal *amo*A were prepared from the plasmid 88exp4 (from the archaeal clones library) and from *Nitrosopumilus maritimus* (obtained from M. Könneke) using the 16S specific primers MCGI-391f (5′-AAGGTTARTCCGAGTGRTTTC) and MCGI-554r (5′-TGACCACTTGAGGTGCTG) for MCGI (Wuchter *et al*., 2006) and the specific archaeal *amo*A primers Arch-*amo*A-for (5′-CTGAYTGGGCYTGGACATC) and Arch-*amo*A-rev (5′-TTCTTCTTTGTTGCCCAGTA) (Wuchter *et al*., 2006), respectively, as described previously (Agogué*et al*., 2008). The other primer set of archaeal *amo*A with the specific primers Arch-*amo*A-for and Arch-*amo*A-rev-New (5′-TTCTTCTTCGTCGCCCAATA) did not produce any PCR product from *N. maritimus*. Consequently, the q-PCR standard was prepared by amplifying a natural deep sea water sample. Both *amoA* PCR products had the same length, as they consisted of the same forward primer and a reverse primer targeting the same location. Based on our hypothesis, we use the term ‘low-ammonia concentration’ archaeal *amo*A (archaeal *amo*A-LAC) for the *amo*A genes obtained with the primer set Arch-*amo*A-for and a modified reverse primer, according to the sequence described by Konstantinidis and colleagues (2009): Arch-*amo*A-rev-New, and ‘high-ammonia concentration’ archaeal *amo*A (archaeal *amo*A-HAC) for that obtained with the primer set Arch-*amo*A-for and Arch-*amo*A-rev (Wuchter *et al*., 2006).

Each amplification was performed under the following conditions: 4 min initial denaturation; 35 cycles at 94°C for 30 s, specific annealing temperature of the primer set for 40 s (61°C for MCGI, 58.5°C for the two archaeal *amo*A primer combinations), 72°C for 2 min, 80°C for 25 s using for 1 U of Pico Maxx high fidelity DNA polymerase (Stratagene), 10× Pico Maxx PCR buffer, 0.25 mM of each dNTP, 8 µg of BSA, 0.2 µM of primers, 3 mM of MgCl_2_ and ultra pure sterile water (Sigma). Amplification products were run on an agarose gel (1%), stained with SYBRGold® (Invitrogen), bands were isolated and purified using the QuickClean 5 M gel extraction kit (GenScript). Purified products were quantified using a Nanodrop® spectrophotometer and the 16S rRNA and *amo*A gene abundance were subsequently calculated from the concentration of the purified DNA and the size fragment. Ten-fold serial dilutions ranging from 10^7^ to 10^0^ gene copies of the corresponding standard were used in triplicate per q-PCR reaction to generate an external quantification standard.

### Q-PCR analysis

All q-PCR analyses were performed on an iCycler iQ 5 thermocycler (Bio-Rad) equipped with i-Cycler iQ software (version 3.1, Bio-Rad). The MCGI 16S rRNA gene abundance, LAC-archaeal *amo*A and HAC-archaeal *amo*A were determined in triplicate on the non-diluted sample and for two different dilutions of the sample (5 times and 25 times diluted). The ‘total’ archaeal *amo*A gene abundance was calculated as the sum of LAC- and HAC-archaeal *amo*A gene abundance assuming specificity of the two primer sets (see Discussion chapter). The reaction mixture (20 µl) contained 1 U of Pico Maxx high fidelity DNA polymerase (Stratagene), 2 µl of 10× Pico Maxx PCR buffer, 0.25 mM of each dNTP, 8 µg of BSA, 0.2 µM of primers, 50 000 times diluted SYBR Green® (Invitrogen) (optimized concentration), a final concentration of 10 nM fluorescein, 3 mM MgCl_2_ and ultra pure sterile water (Sigma). All reactions were performed in 96-well q-PCR plates (Bio-Rad) with optical tape (Bio-Rad). One microlitre of diluted or non-diluted environmental DNA was added to 19 µl of mix in each well. Accumulation of newly amplified double stranded gene products was followed online as the increase of fluorescence due to the binding of the fluorescent dyes SYBRGreen® and fluorescein. Specificity of the q-PCR reaction was tested on agarose gel electrophoresis and with a melting curve analysis (60–94°C) in order to identify unspecific PCR products. PCR efficiencies and correlation coefficients for standard curves were as follows: for the MCGI 16S rRNA gene assay, 87.9–105.8% and *r*^2^ = 0.991–0.999, for the archaeal *amo*A-HAC assay, 85.3–117% and *r*^2^ = 0.981–0.999 and for the archaeal *amo*A-LAC assay, 79.7–122% and *r*^2^ = 0.993–0.999. Each gene fragment was detected using a standard for the specific quantification of MCGI 16S rRNA gene abundance, archaeal *amo*A-LAC and *amo*A-HAC genes and primer combinations and annealing temperature as described above. Thermocycling was performed as follows: initial denaturation at 95°C for 4 min; amplification: 41 cycles, at 95°C for 30 s, primer annealing temperature for 40 s, and extension at 72°C for 30 s, 80°C for 25 s, with a plate read between each cycle; melting curve 60–94°C with a read every 0.5°C held for 1 s between each read.

### Primer specificity

The specificity of the two primer sets used in this study was tested on full-length archaeal *amo*A clones. The full-length archaeal *amo*A from different samples was amplified using the primers cren amo_F (5′-ATGGTCTGGCTAAGACGMTGTA) (Hallam *et al*., 2006b) and amoAR (5′-GCGGCCATCCATCTGTATGT) (Francis *et al*., 2005). Thermocycling was performed as follows: initial denaturation at 94°C for 4 min; amplification: 35 cycles, at 94°C for 1 min, 55°C for 1 min, and extension at 72°C for 1 min, followed by a final extension step at 72°C for 7 min and holding at 4°C. The PCR product was purified using PCRExtract MiniKit (5-PRIME) and cloned with the TOPO-TA cloning kit® (Invitrogen) according to the manufacturer's instructions. Clones were checked for the right insert by running the PCR product on a 2% agarose gel. Sequencing was performed by MACROGEN Europe using the M13 primers. The sequence data were compiled and aligned using MEGA-4 software. A total of 35 clones belonging to the two clusters (HAC or LAC), containing 0–4 mismatches as related to the sequence of the two different primer sets were selected. Archaeal *amo*A from these selected clones was amplified and purified as described above. Purified products were quantified using a Nanodrop® spectrophotometer and the gene abundance of the *amo*A genes were subsequently calculated from the concentration of the purified DNA and the size fragment. Three dilutions were prepared: 10^2^, 10^4^ and 10^6^ copies µl^−1^. The abundance of the *amo*A gene was quantified with the 2 sets of primers following the protocol previously described above for all the clones at the higher concentration (10^6^ µl^−1^) and for selected clones at the two lower gene concentrations (10^4^ and 10^2^ µl^−1^). The percentage of abundance of *amo*A belonging to the HAC cluster determined with the LAC primer set was compared with its abundance determined with the HAC primer set and vice versa.

Additionally, six DNA mixtures were prepared with clones belonging to HAC- and LAC-AOA clusters with proportions ranging from 0.2% of one clone + 99.8% of the other clone to ∼ 50% of each clone. The abundance of the HAC- and LAC-AOA was then measured in the mixture with the corresponding primer set, and the proportion of HAC- and LAC-*amo*A gene calculated.

### Cloning, sequencing and phylogenetic analysis of archaeal *amo*A

The archaeal *amo*A was amplified with the two sets of primers under the same conditions as described above for q-PCR. The PCR product was purified with QuickClean 5 M PCR purification Kit (Genscript) and cloned with the TOPO-TA cloning kit® (Invitrogen) according to the manufacturer's instructions. Clones were checked for the right insert by running the PCR product on a 2% agarose gel. Sequencing was performed by MACROGEN (Korea) using the M13 primers. The sequence data were compiled using MEGA-4 software, and aligned together with environmental archaeal *amo*A sequences, full-length sequences of *amo*A genes from *Nitrosopumilus maritimus* and *Candidatus* Nitrosocaldus yellowstonii obtained from the NCBI database. Phylogenetic analyses were conducted in MEGA-4 (Tamura *et al*., 2007). The evolutionary history was inferred using the neighbour-joining method (Saitou and Nei, 1987). The bootstrap consensus tree inferred from 1000 replicates is taken to represent the evolutionary history of the taxa analysed (Felsenstein, 1985). Branches corresponding to partitions reproduced in less than 50% bootstrap replicates are collapsed. The tree is drawn to scale, with branch lengths in the same units as those of the evolutionary distances used to infer the phylogenetic tree. The evolutionary distances were computed using the Maximum Composite Likelihood method (Tamura *et al*., 2004) and are in the units of the number of base substitutions per site. All positions containing gaps and missing data were eliminated from the dataset (complete deletion option). There were a total of 210 positions in the final dataset. Phylogenetic trees were drawn using iTOL (Letunic and Bork, 2007).

Rarefaction analysis was performed using DOTUR (Schloss and Handelsman, 2005) for each sample and depth layer to compare the archaeal *amo*A richness within each clone library for both sets of primers. Operational taxonomic units were defined as a group of sequences differing by less than 2%. The Chao and ACE richness index and the Shannon and Simpson diversity index were also obtained for the different clone libraries using DOTUR.

Sequence information obtained in this study has been deposited in GenBank, accession numbers HM449165 to HM449449.

### Statistical analyses

Spearman rank correlation was performed to analyse the relations between several parameters. Correlation analyses were performed with SigmaPlot 11.00 (Systat Software). Comparison of the phylogenetic composition of the AOA between the different environments was conducted in UniFrac (Lozupone and Knight, 2005; Lozupone *et al*., 2006; Hamady *et al*., 2010).

## Supporting information

**Fig. S1.** Location of the two sampling areas:(A) the tropical Atlantic and (B) stations occupied in the coastalArctic off Ny Ålesund (Kongsfjorden, Spitsbergen, Norway).

**Fig. S2.** Depth profile of prokaryotic abundance (PA) and heterotrophic activity (PHA, measured via leucine incorporation) obtained in (A) the coastal Arctic and (B) the tropical Atlantic. (C) Dark dissolved inorganic carbon fixation (DIC) in the water column of the coastal Arctic and tropical Atlantic.

**Fig. S3.** Proportion of HAC- or LAC- clonesadded to a DNA mixture as compared with the proportion of HAC orLAC-*amoA* measured with the corresponding primer in the mixture.

**Fig. S4.** Distribution of archaeal *amoA*copy numbers along the Romanche Fracture Zone of the tropicalAtlantic (see Fig. S1 for sample location and stations) obtainedwith (A) the ‘high-ammonia concentration’ primer set,(B) the ‘low-ammonia concentration’ primer set and (C)the ratio between archaeal *amoA* copy numbers determined withthe ‘high’ versus ‘low ammoniaconcentration’ primer sets (1:1 line is indicated at around 100 m depth).

**Fig. S5.** Distribution of (A) abundance ofMarine Crenarcheaota Group I (MCGI) genes along a transect throughthe Romanche Fracture Zone in the tropical Atlantic and the ratioarchaeal *amoA* to MCGI obtained with (B) the ‘highammonia concentration’ and (C) the ‘low ammoniaconcentration’ primer set.

**Fig. S6.** Depth profile of the ratio betweentotal archaeal *amoA* (sum of HAC- and LAC-*amoA*) and16S rRNA of MCGI for the Arctic (open circles) and the Atlantic(full circles).

**Fig. S7**. Rarefaction curves for the archaeal*amo*A gene obtained with the ‘high ammoniaconcentration’ (HAC) and the ‘low ammoniaconcentration’ (LAC) primer sets at 100, 250, 1750 and 7000 m depth in the tropical Atlantic.

**Fig. S8.** Scatter plot for the first twoprincipal components (PCA) obtained for the phylogeneticcomposition with the ‘high-ammonia concentration’ (HAC)and ‘low-ammonia concentration’ (LAC) primer set at different depth layers in the Romanche Fracture Zone of the tropical Atlantic (UniFrac analysis). Numbers next to dots indicate depth in m of the sample.

**Fig. S9.** Ammonium (A, C) and nitrite (B, D) concentration throughout the water column in the Arctic (A, B) and Atlantic (C, D).

**Table S1.** Median and range (max–min) ofthe percentage of recovery of archaeal *amo*A originating fromclones belonging to the ‘high ammonia concentration’(HAC) and the ‘low ammonia concentration’ (LAC) cluster as measured with the two primer sets used in this study.

**Table S2.** Depth-averaged concentrations (inμM) of ammonium (NH_4_^+^), nitrite(NO_2_^-^) and nitrate(NO_3_^-^) and the abundance of crenarchaealgenes (HAC *amo*A, LAC *amo*A, total *amo*A andMCGI), and the corresponding ratios obtained in the coastalArctic.

**Table S3.** Depth-averaged concentrations (inμM) of ammonium (NH_4_^+^), nitrite(NO_2_^-^), nitrate (NO_3_^-^)and oxygen (O_2_) and the abundance of crenarchaeal genes(HAC *amo*A, LAC *amo*A, total *amo*A and MCGI), andthe corresponding ratios obtained in the tropical Atlantic.

**Table S4.** Clones of the NCBI database (as for30 March 2010) containing the exact sequence from the reverseprimer of the LAC-*amo*A or the HAC-*amo*A and theirorigin.
