# Supplementary figures and images for: Archaeal amoA gene diversity points to distinct biogeography of ammonia-oxidizing Crenarchaeota in the ocean
Source: Environ Microbiol. 2013 May;15(5):1647–58. doi: 10.1111/j.1462-2920.2012.02801.x (PMC3712475; doi:10.1111/j.1462-2920.2012.02801.x)

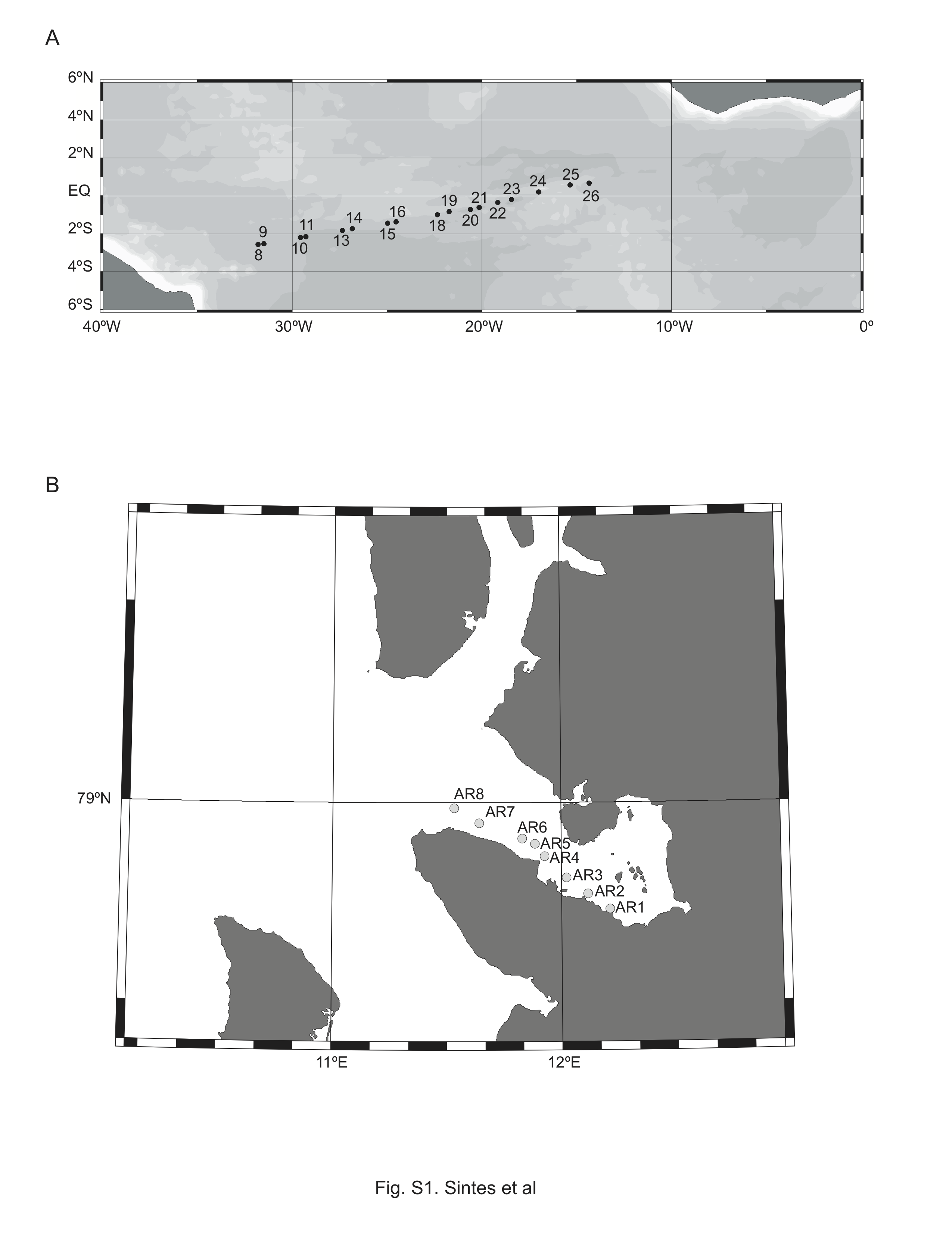

Supplement: Supplementary file 1 [file emi0015-1647-SD1.tiff]

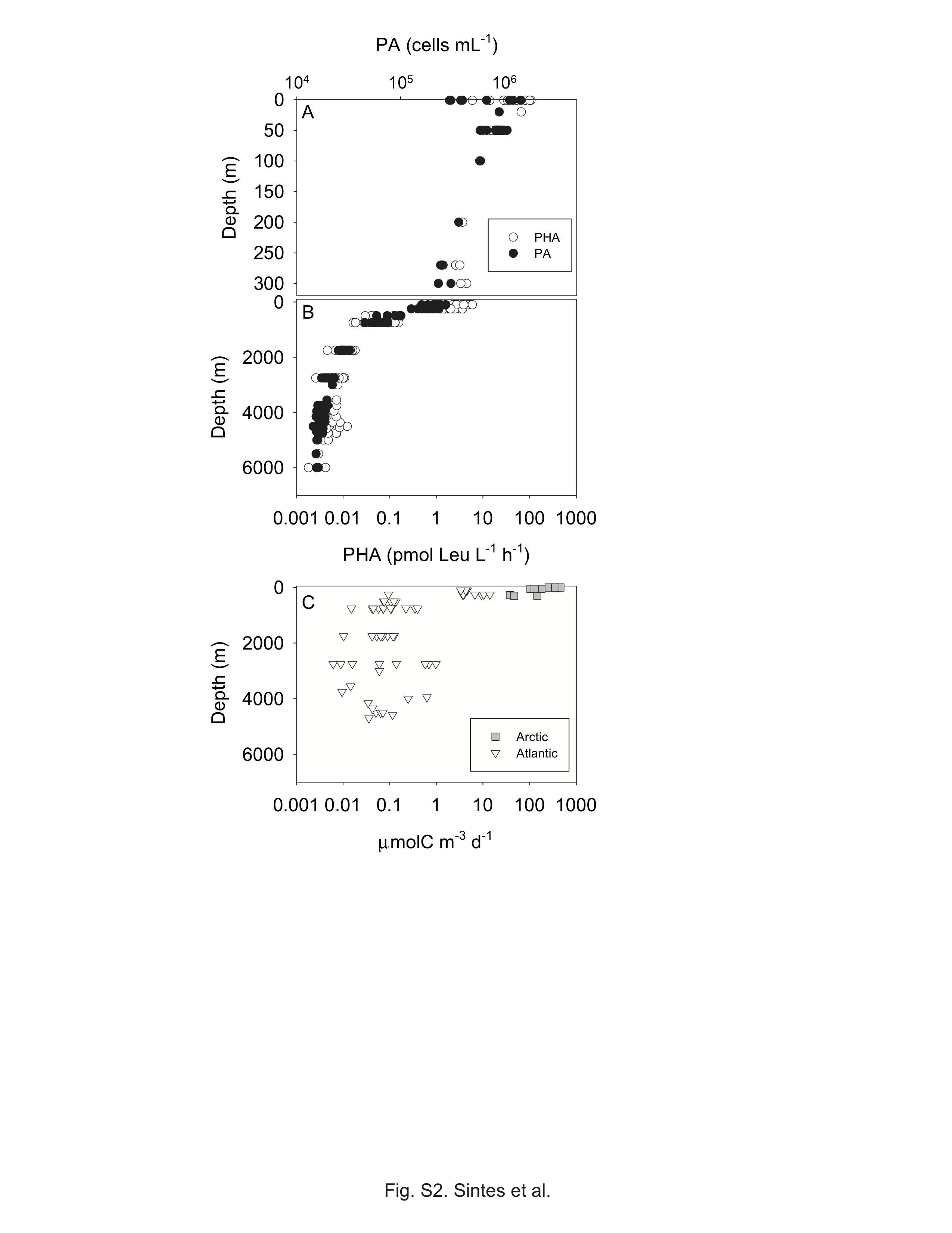

Supplement: Supplementary file 2 [file emi0015-1647-SD2.tiff]

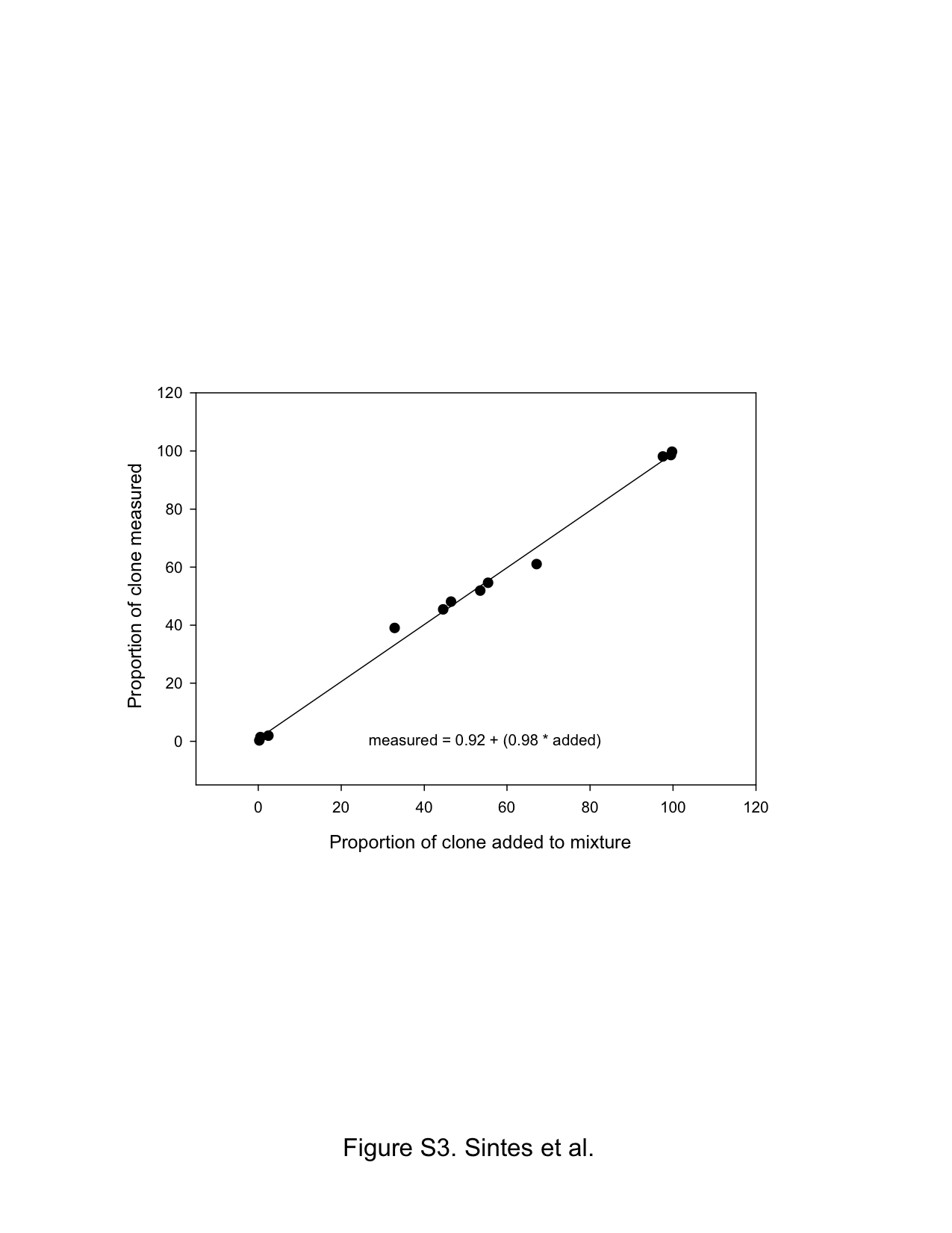

Supplement: Supplementary file 3 [file emi0015-1647-SD3.tiff]

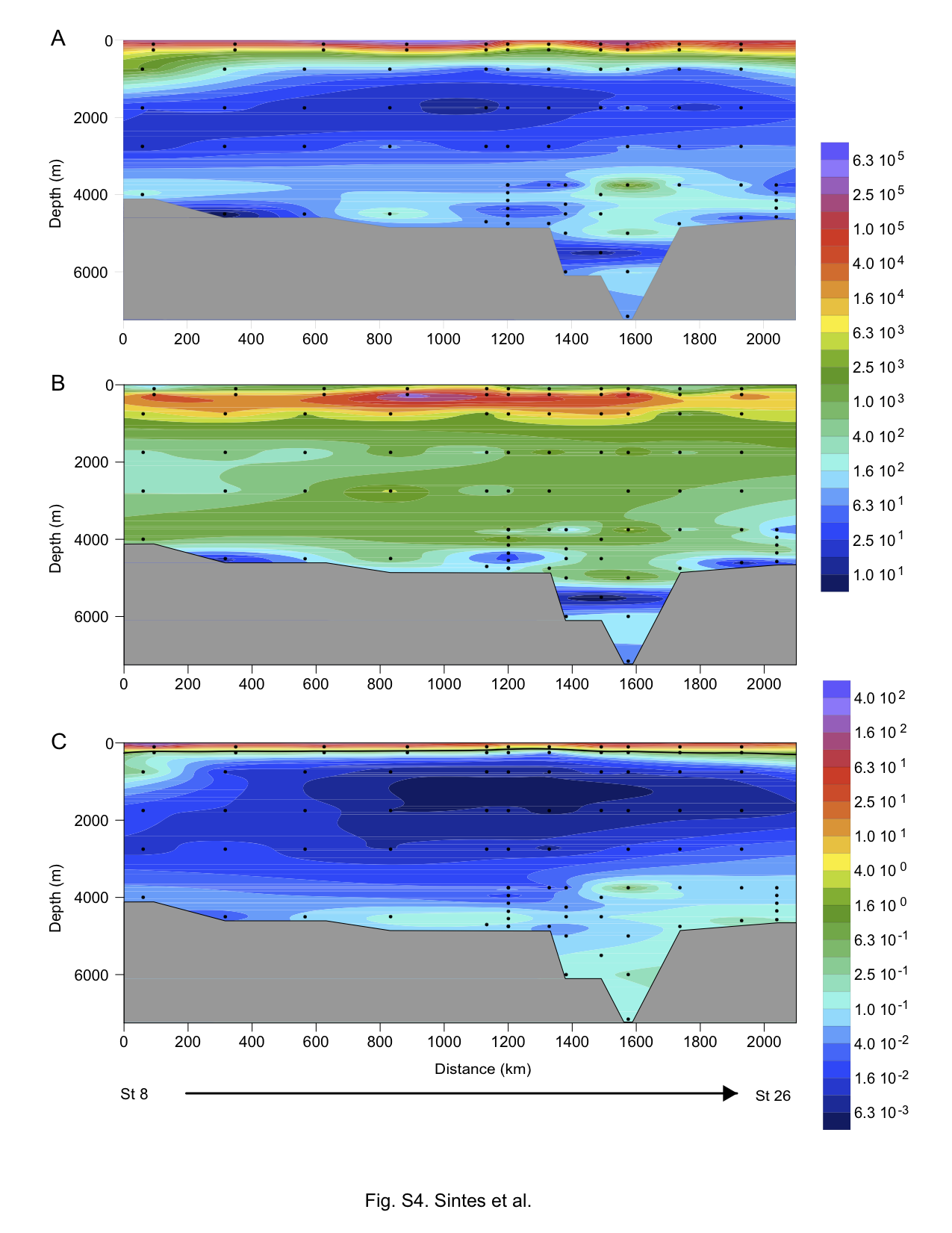

Supplement: Supplementary file 4 [file emi0015-1647-SD4.tiff]

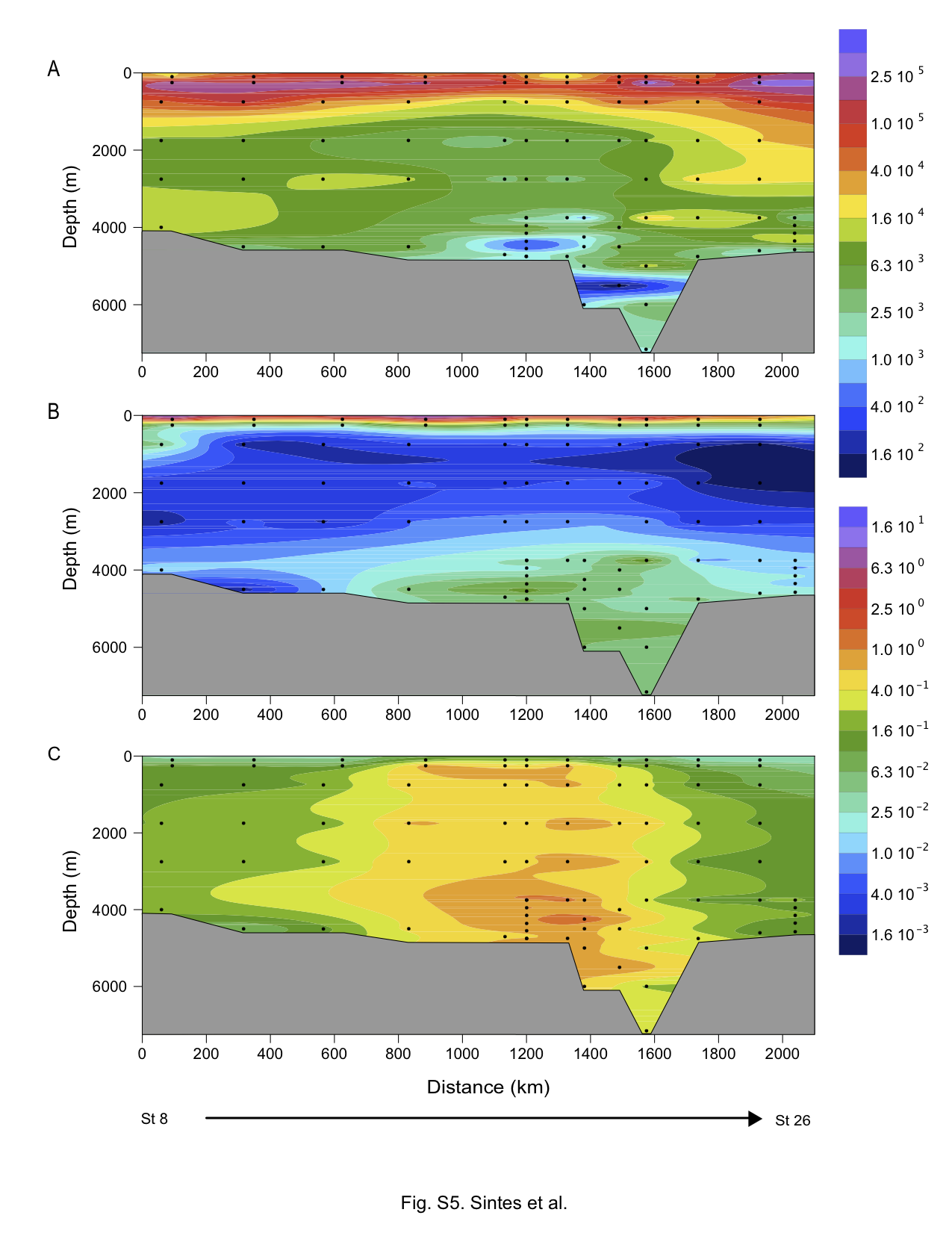

Supplement: Supplementary file 5 [file emi0015-1647-SD5.tiff]

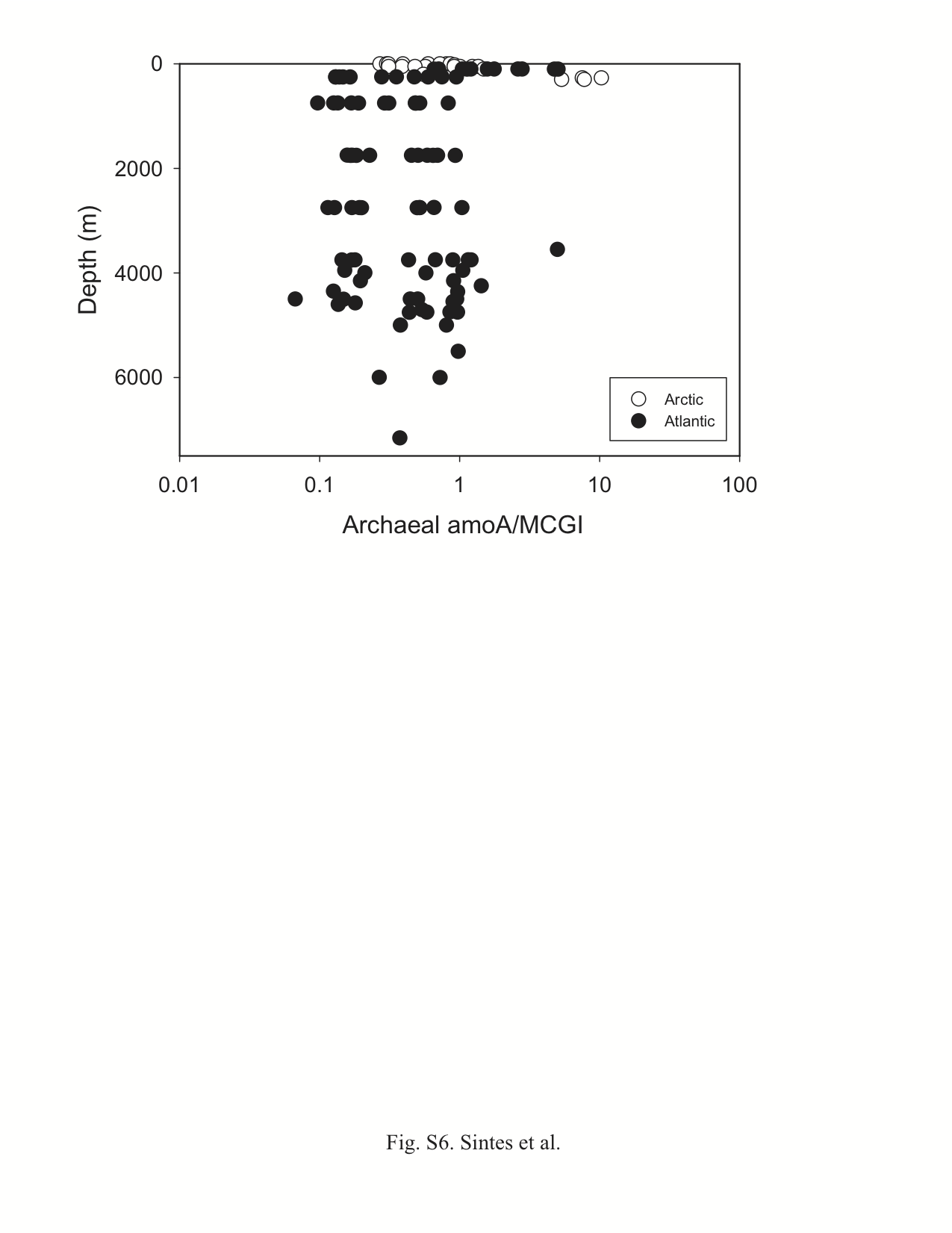

Supplement: Supplementary file 6 [file emi0015-1647-SD6.tiff]

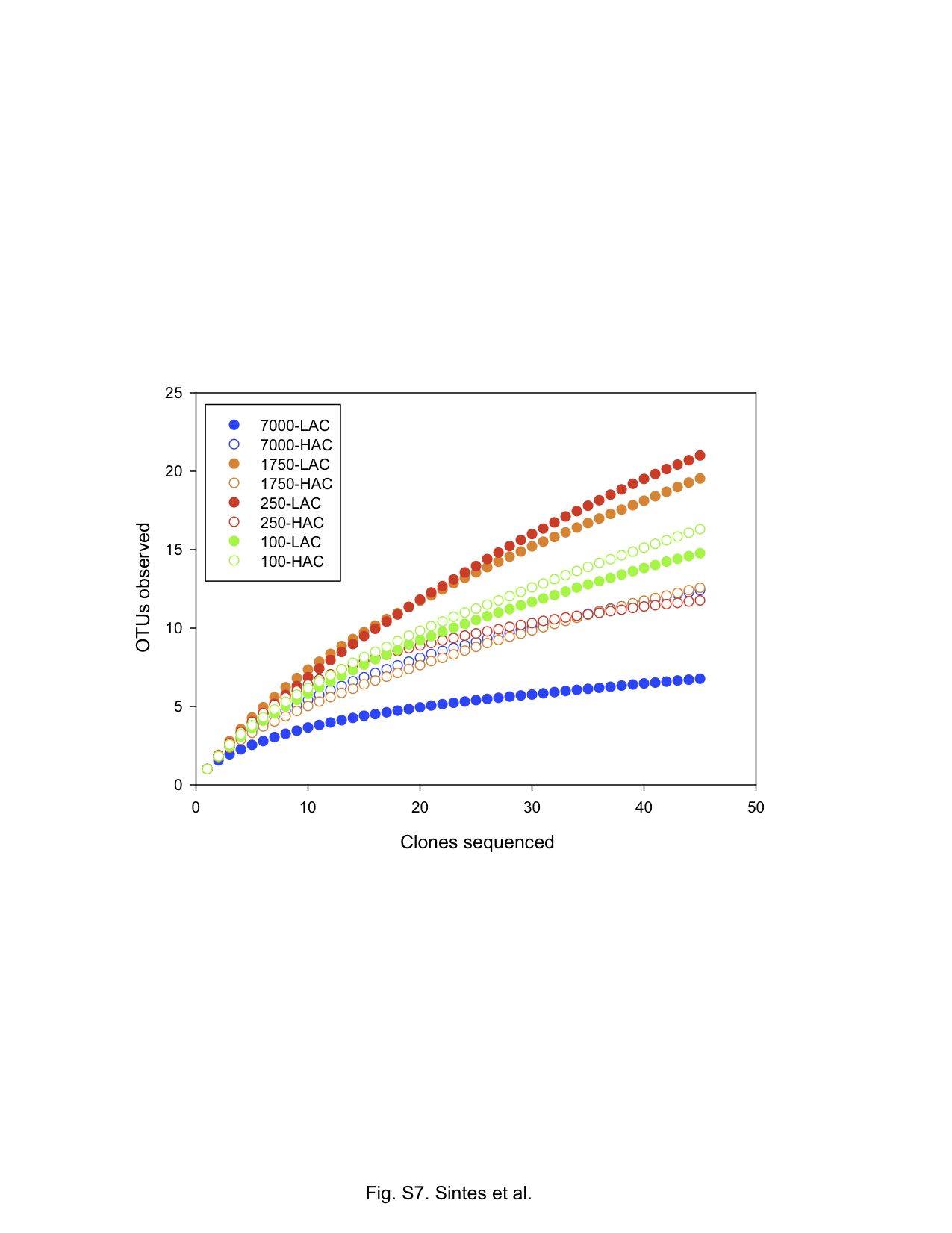

Supplement: Supplementary file 7 [file emi0015-1647-SD7.tiff]

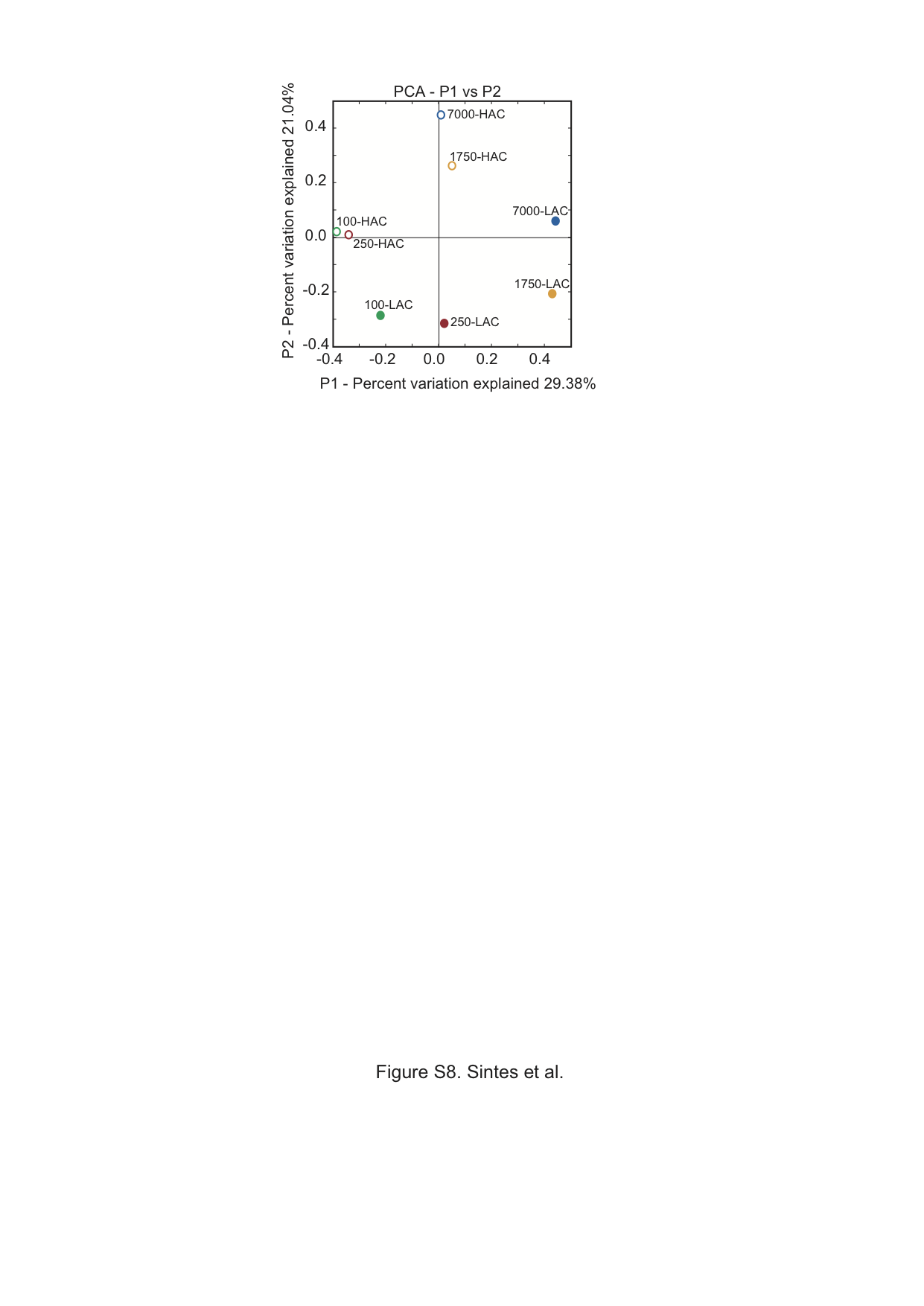

Supplement: Supplementary file 8 [file emi0015-1647-SD8.tiff]

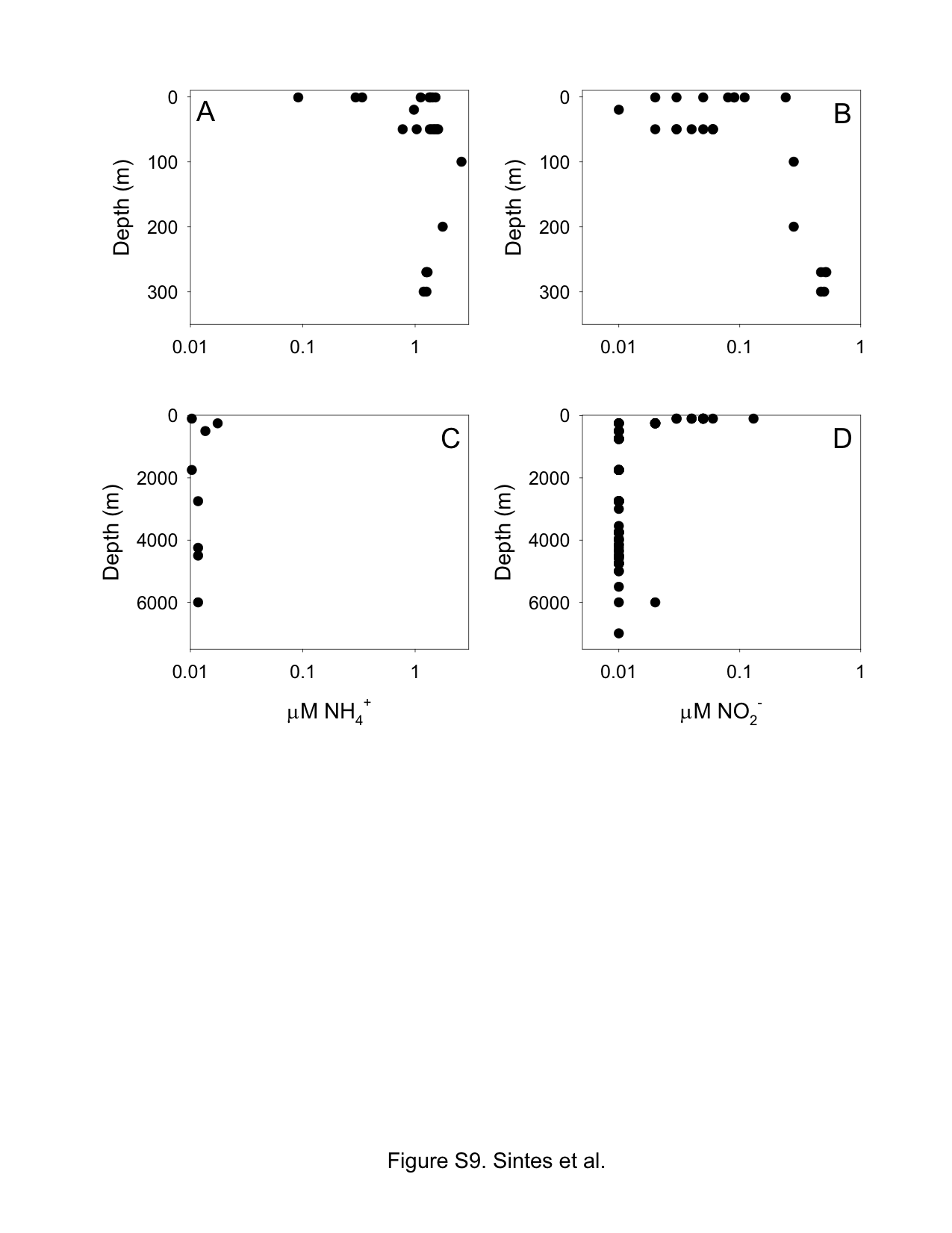

Supplement: Supplementary file 9 [file emi0015-1647-SD9.tiff]
