# Supplementary material for: Archaeal amoA gene diversity points to distinct biogeography of ammonia-oxidizing Crenarchaeota in the ocean
Source: Environ Microbiol. 2013 May;15(5):1647–58. doi: 10.1111/j.1462-2920.2012.02801.x (PMC3712475; doi:10.1111/j.1462-2920.2012.02801.x)
Supplement: Supplementary file 10 [file emi0015-1647-SD10.doc]

Table S1. Median and range (max-min) of the percentage of recovery of archaeal *amo*A originating from clones belonging to the ‘high ammonia concentration’ (HAC) and the ‘low ammonia concentration’ (LAC) cluster as measured with the two primer sets used in this study. The number of different clones tested at different concentrations of *amo*A per µL of DNA extract is indicated (n).

|  | HAC-*amo*A 10^6^ µL^-1^ | HAC-*amo*A 10^4^ µL^-1^ | HAC-*amo*A 10^2^ µL^-1^ |
| --- | --- | --- | --- |
| Amplified with LAC primer set | 0.04  (0.00-1.96)  n = 11 | 0.76  (0.47-2.64)  n = 3 | 1.22  (0.04-6.54)  n = 4 |
|  | LAC-*amo*A 10^6^ µL^-1^ | LAC-*amo*A 10^4^ µL^-1^ | LAC-*amo*A 10^2^ µL^-1^ |
| Amplified with HAC primer set | 1.59  (0.47-8.98)  n = 24 | 0.64  (0.06-1.22)  n = 5 | 0.99  (0.01-6.91)  n = 13 |
