# Supplementary material for: Archaeal amoA gene diversity points to distinct biogeography of ammonia-oxidizing Crenarchaeota in the ocean
Source: Environ Microbiol. 2013 May;15(5):1647–58. doi: 10.1111/j.1462-2920.2012.02801.x (PMC3712475; doi:10.1111/j.1462-2920.2012.02801.x)
Supplement: Supplementary file 11 [file emi0015-1647-SD11.doc]

**Table S2.** Depth-averaged concentrations (in µM) of ammonium (NH_4_^+^), nitrite (NO_2_^-^) and nitrate (NO_3_^-^) and the abundance of crenarchaeal genes (HAC *amo*A, LAC *amo*A, total *amo*A and MCGI), and the corresponding ratios obtained in the coastal Arctic. HAC – high-ammonia concentration; LAC – low-ammonia concentration, MCGI – marine Crenarchaeota Group I.

|  | 1-20 m | 50 m | 100-300 m |
| --- | --- | --- | --- |
| NH_4_^+^ | 0.942 | 1.186 | 1.515 |
| NO_2_^-^ | 0.079 | 0.039 | 0.430 |
| NO_3_^-^ | 0.428 | 0.438 | 8.167 |
| HAC *amo*A genes mL^-1^ | 15 | 161 | 7919 |
| LAC *amo*A genes mL^-1^ | 0.01 | 0.10 | 0.49 |
| Total archaeal *amo*A genes mL^-1^ | 15 | 162 | 7919 |
| HAC/LAC *amo*A | 7209 | 1818 | 14854 |
| MCGI genes mL^-1^ | 25 | 180 | 1482 |
| HAC *amo*A/MCGI | 0.57 | 0.78 | 8.08 |
| LAC *amo*A/MCGI | 0.0004 | 0.0016 | 0.0006 |
| Total archaeal *amo*A/MCGI | 0.57 | 0.78 | 8.08 |
