# Supplementary material for: Archaeal amoA gene diversity points to distinct biogeography of ammonia-oxidizing Crenarchaeota in the ocean
Source: Environ Microbiol. 2013 May;15(5):1647–58. doi: 10.1111/j.1462-2920.2012.02801.x (PMC3712475; doi:10.1111/j.1462-2920.2012.02801.x)
Supplement: Supplementary file 12 [file emi0015-1647-SD12.doc]

**Table S3. Depth-averaged concentrations (in µM) of ammonium (NH_4_^+^), nitrite (NO_2_^-^), nitrate (NO_3_^-^) and oxygen (O_2_) and the abundance of crenarchaeal genes (HAC *amo*A, LAC *amo*A, total *amo*A and MCGI), and the corresponding ratios obtained in the tropical Atlantic. HAC – high ammonia concentration; LAC – low ammonia concentration, MCGI – marine Crenarchaeota Group I.**

|  |  | 31.6ºW | 29.4ºW | 27.1ºW | 24.7ºW | 22.3ºW | 21.7ºW | 20.3ºW | 19ºW | 18.4ºW | 17.0ºW | 14.5ºW |  |
| --- | --- | --- | --- | --- | --- | --- | --- | --- | --- | --- | --- | --- | --- |
|  |  | St 8/9 | St 10/11 | St 13/14 | St 15/16 | St 18 | St19 | St 20/21 | St 22 | St23 | St 24 | St 25/26 | Average |
| 100m | NH_4_^+^ |  | 0.01 | <0.01 | <0.01 | <0.01 | <0.01 | <0.01 | <0.01 | <0.01 | <0.01 | <0.01 | n.d. |
|  | NO_2_^-^ | 0.133 | 0.059 | 0.046 | 0.035 | 0.046 | 0.050 | 0.054 | 0.050 | 0.035 | 0.034 | 0.028 | 0.052 |
|  | NO_3_^-^ | 20.15 | 20.79 | 22.09 | 19.41 | 12.59 | 11.83 | 11.72 | 11.03 | 11.74 | 13.53 | 16.16 | 15.55 |
|  | O_2_ | 140 | 111 | 101 | 117 | 164 | 168 | 169 | 170 | 166 | 152 | 127 | 144 |
|  | HAC *amo*A genes mL^-1^ | 118078 | 89507 | 155880 | 272167 | 220667 | 26910 | 14138 | 136400 | 202200 | 29900 | 104550 | 124581 |
|  | LAC *amo*A genes mL^-1^ | 983 | 3808 | 3798 | 9610 | 17033 | 6426 | 2711 | 5158 | 6474 | 1504 | 4950 | 5678 |
|  | Total archaeal *amo*A genes mL^-1^ | 119061 | 93315 | 159678 | 281777 | 237700 | 33336 | 16849 | 141558 | 208674 | 31404 | 109500 | 130259 |
|  | HAC/LAC *amo*A | 120.11 | 23.50 | 41.04 | 28.32 | 12.95 | 4.19 | 5.21 | 26.44 | 31.23 | 19.88 | 21.12 | 30.365 |
|  | MCGI genes mL^-1^ | 23538 | 77700 | 90300 | 59263 | 84900 | 31937 | 14968 | 89776 | 80040 | 44373 | 166800 | 69418 |
|  | HAC *amo*A/MCGI | 5.02 | 1.15 | 1.73 | 4.59 | 2.60 | 0.84 | 0.94 | 1.52 | 2.53 | 0.67 | 0.63 | 2.020 |
|  | LAC *amo*A/MCGI | 0.04 | 0.05 | 0.04 | 0.16 | 0.20 | 0.20 | 0.18 | 0.06 | 0.08 | 0.03 | 0.03 | 0.10 |
|  | Total archaeal *amo*A/MCGI | 5.06 | 1.20 | 1.77 | 4.75 | 2.80 | 1.04 | 1.13 | 1.58 | 2.61 | 0.71 | 0.66 | 2.12 |
| 250m | NH_4_^+^ |  | <0.01 | <0.01 | <0.01 | 0.02 | 0.01 | <0.01 | <0.01 | <0.01 | <0.01 |  | n.d. |
|  | NO_2_^-^ | 0.015 | 0.018 | 0.018 | 0.015 | 0.015 | 0.021 | 0.012 | 0.018 | 0.019 | 0.022 |  | 0.017 |
|  | NO_3_^-^ | 26.28 | 24.20 | 27.51 | 26.53 | 26.03 | 27.80 | 29.43 | 27.88 | 27.43 | 25.45 |  | 26.85 |
|  | O_2_ | 91 | 106 | 89 | 97 | 100 | 93 | 85 | 84 | 88 | 95 | 96 | 93 |
|  | HAC *amo*A genes mL^-1^ | 8064 | 5417 | 5274 | 27880 | 9923 | 3812 | 1993 | 8765 | 30780 | 6038 | 20627 | 11688 |
|  | LAC *amo*A genes mL^-1^ | 33603 | 15903 | 21600 | 137040 | 54110 | 27510 | 41223 | 16758 | 76200 | 6057 | 19977 | 40907 |
|  | Total archaeal *amo*A genes mL^-1^ | 41667 | 21320 | 26874 | 164920 | 64033 | 31322 | 43216 | 25523 | 106980 | 12094 | 40603 | 52596 |
|  | HAC/LAC *amo*A | 0.240 | 0.341 | 0.244 | 0.203 | 0.183 | 0.139 | 0.048 | 0.523 | 0.404 | 0.997 | 1.033 | 0.396 |
|  | MCGI genes mL^-1^ | 252733 | 154700 | 206520 | 174400 | 107590 | 66150 | 57846 | 72308 | 385800 | 82570 | 312433 | 170277 |
|  | HAC *amo*A/MCGI | 0.032 | 0.035 | 0.026 | 0.160 | 0.092 | 0.058 | 0.034 | 0.121 | 0.080 | 0.073 | 0.066 | 0.071 |
|  | LAC *amo*A/MCGI | 0.13 | 0.10 | 0.10 | 0.79 | 0.50 | 0.42 | 0.71 | 0.23 | 0.20 | 0.07 | 0.06 | 0.30 |
|  | Total archaeal *amo*A/MCGI | 0.16 | 0.14 | 0.13 | 0.95 | 0.60 | 0.47 | 0.75 | 0.35 | 0.28 | 0.15 | 0.13 | 0.37 |
| 750m | NH_4_^+^ |  | <0.01 | <0.01 | 0.01 | <0.01 | <0.01 | <0.01 | <0.01 | <0.01 | <0.01 | <0.01 | n.d. |
|  | NO_2_^-^ | 0.009 | 0.006 | 0.006 | 0.006 | 0.004 | 0.008 | 0.007 | 0.013 | 0.015 | 0.011 | 0.007 | 0.008 |
|  | NO_3_^-^ | 33.20 | 33.36 | 33.40 | 34.09 | 34.32 | 34.18 | 34.42 | 33.96 | 34.22 | 34.02 | 34.31 | 33.95 |
|  | O_2_ | 166 | 160 | 162 | 149 | 148 | 146 | 147 | 148 | 145 | 146 | 146 | 151 |
|  | HAC *amo*A genes mL^-1^ | 2442 | 211 | 95 | 110 | 37 | 106 | 76 | 266 | 159 | 49 | 117 | 333 |
|  | LAC *amo*A genes mL^-1^ | 7896 | 15657 | 5976 | 14271 | 4655 | 11421 | 12941 | 17920 | 16208 | 3139 | 8461 | 10777 |
|  | Total archaeal *amo*A genes mL^-1^ | 10338 | 15868 | 6071 | 14380 | 4691 | 11527 | 13018 | 18186 | 16367 | 3187 | 8578 | 11110 |
|  | HAC/LAC *amo*A | 0.309 | 0.013 | 0.016 | 0.008 | 0.008 | 0.009 | 0.006 | 0.015 | 0.010 | 0.016 | 0.014 | 0.038 |
|  | MCGI genes mL^-1^ | 54600 | 117700 | 36090 | 27728 | 9731 | 23800 | 15732 | 58400 | 56320 | 25337 | 88760 | 46745 |
|  | HAC *amo*A/MCGI | 0.045 | 0.002 | 0.003 | 0.004 | 0.004 | 0.004 | 0.005 | 0.005 | 0.003 | 0.002 | 0.001 | 0.007 |
|  | LAC *amo*A/MCGI | 0.14 | 0.13 | 0.17 | 0.51 | 0.48 | 0.48 | 0.82 | 0.31 | 0.29 | 0.12 | 0.10 | 0.32 |
|  | Total archaeal *amo*A/MCGI | 0.19 | 0.13 | 0.17 | 0.52 | 0.48 | 0.48 | 0.83 | 0.31 | 0.29 | 0.13 | 0.10 | 0.33 |
| 1750m | NH_4_^+^ |  | 0.01 | <0.01 | 0.01 | <0.01 | <0.01 | <0.01 | <0.01 | <0.01 |  |  | n.d. |
|  | NO_2_^-^ |  | 0.015 | 0.008 | 0.000 | 0.007 | 0.006 | 0.003 | 0.012 | 0.011 | 0.009 | 0.008 | 0.008 |
|  | NO_3_^-^ |  | 19.53 | 19.28 | 19.85 | 19.95 | 19.84 | 20.09 | 19.82 | 19.74 | 19.84 | 20.21 | 19.81 |
|  | O_2_ | 246 | 244 | 248 | 243 | 242 | 241 | 241 | 241 | 240 | 239 | 235 | 242 |
|  | HAC *amo*A genes mL^-1^ | 25 | 32 | 16 | 21 | 9 | 25 | 24 | 19 | 63 | 20 | 28 | 26 |
|  | LAC *amo*A genes mL^-1^ | 1445 | 2109 | 1075 | 3451 | 1361 | 2973 | 5079 | 2335 | 6816 | 2076 | 3909 | 2966 |
|  | Total archaeal *amo*A genes mL^-1^ | 1470 | 2142 | 1091 | 3472 | 1370 | 2999 | 5103 | 2354 | 6879 | 2096 | 3937 | 2992 |
|  | HAC/LAC *amo*A | 0.017 | 0.015 | 0.015 | 0.006 | 0.007 | 0.009 | 0.005 | 0.008 | 0.009 | 0.010 | 0.007 | 0.010 |
|  | MCGI genes mL^-1^ | 8864 | 9422 | 5967 | 4998 | 2716 | 6630 | 5493 | 4012 | 10658 | 12264 | 25047 | 8734 |
|  | HAC *amo*A/MCGI | 0.003 | 0.003 | 0.003 | 0.004 | 0.003 | 0.004 | 0.004 | 0.005 | 0.006 | 0.002 | 0.001 | 0.003 |
|  | LAC *amo*A/MCGI | 0.16 | 0.22 | 0.18 | 0.69 | 0.50 | 0.45 | 0.92 | 0.58 | 0.64 | 0.17 | 0.16 | 0.43 |
|  | Total archaeal *amo*A/MCGI | 0.17 | 0.23 | 0.18 | 0.69 | 0.50 | 0.45 | 0.93 | 0.59 | 0.65 | 0.17 | 0.16 | 0.43 |
| 2750-7000 m | NH_4_^+^ |  | <0.01 | <0.01 | <0.01 | <0.01 | <0.01 | <0.01 | <0.01 | <0.01 |  |  | n.d. |
|  | NO_2_^-^ | 0.009 | 0.015 | 0.008 | 0.008 | 0.005 | 0.008 | 0.010 | 0.010 | 0.012 | 0.008 | 0.010 | 0.009 |
|  | NO_3_^-^ | 20.86 | 21.48 | 25.01 | 24.94 | 24.81 | 26.95 | 24.65 | 24.80 | 23.49 | 23.08 | 23.40 | 23.95 |
|  | O_2_ | 250 | 237 | 236 | 235 | 235 | 271 | 237 | 236 | 238 | 238 | 238 | 241 |
|  | HAC *amo*A genes mL^-1^ | 102 | 22 | 39 | 190 | 60 | 80 | 61 | 187 | 1697 | 90 | 89 | 238 |
|  | LAC *amo*A genes mL^-1^ | 2348 | 830 | 1522 | 4995 | 1259 | 2293 | 1618 | 2027 | 5315 | 1660 | 1230 | 2282 |
|  | Total archaeal *amo*A genes mL^-1^ | 2450 | 852 | 1561 | 5185 | 1319 | 2372 | 1679 | 2214 | 7012 | 1750 | 1182 | 2507 |
|  | HAC/LAC *amo*A | 0.035 | 0.028 | 0.043 | 0.061 | 0.059 | 0.073 | 0.047 | 0.100 | 0.233 | 0.066 | 0.103 | 0.077 |
|  | MCGI genes mL^-1^ | 12537 | 5534 | 8470 | 8634 | 2514 | 2744 | 1699 | 4329 | 16443 | 11611 | 8054 | 7506 |
|  | HAC *amo*A/MCGI | 0.007 | 0.003 | 0.006 | 0.029 | 0.029 | 0.057 | 0.041 | 0.062 | 0.067 | 0.018 | 0.014 | 0.030 |
|  | LAC *amo*A/MCGI | 0.18 | 0.13 | 0.17 | 0.55 | 0.50 | 0.91 | 0.95 | 0.60 | 0.37 | 0.21 | 0.13 | 0.43 |
|  | Total archaeal *amo*A/MCGI | 0.19 | 0.13 | 0.17 | 0.58 | 0.53 | 0.96 | 0.99 | 0.66 | 0.44 | 0.23 | 0.14 | 0.46 |
