# Supplementary material for: Archaeal amoA gene diversity points to distinct biogeography of ammonia-oxidizing Crenarchaeota in the ocean
Source: Environ Microbiol. 2013 May;15(5):1647–58. doi: 10.1111/j.1462-2920.2012.02801.x (PMC3712475; doi:10.1111/j.1462-2920.2012.02801.x)
Supplement: Supplementary file 13 [file emi0015-1647-SD13.doc]

**Table S4. Clones of the NCBI database (as for 30 March 2010) containing the exact sequence from the reverse primer of the LAC-*amo*A or the HAC-*amo*A and their origin. Predominance of clones obtained with either primer set is presented in bold. HAC – ‘high ammonia concentration’; LAC – ‘low ammonia concentration’.**

|  | **Number of clones** | **Number of clones** | **Environment** | **Depth** | **Reference** |
| --- | --- | --- | --- | --- | --- |
|  | **LAC-amoA** | **HAC-amoA** |  |  |  |
| **Seawater** | 8 | **18** | Coastal Arctic ocean | ? | Christman et al., unpublished |
|  | **7** | 6 | Arctic ocean | 235 m | (Kalanetra et al., 2009) |
|  | 0 | **17** | Arctic ocean | 131 m | (Kalanetra et al., 2009) |
|  | 0 | **17** | Arctic ocean | 55 m | (Kalanetra et al., 2009) |
|  | 2 | 2 | Antartic coastal | CDW (149-661 m) | (Kalanetra et al., 2009) |
|  | 0 | **2** | Antartic coastal | WW (46-103 m) | (Kalanetra et al., 2009) |
|  | 0 | **2** | Antartic coastal | SSW (1-13) | (Kalanetra et al., 2009) |
|  | 0 | **7** | Antartic coastal | Unspecified | (Kalanetra et al., 2009) |
|  | **1** | 0 | North Pacific Subtropical Gyre 22.75 N 158.0 W | 4000 m | (Konstantinidis and DeLong, 2008) |
|  | 2 | **3** | eastern tropical north pacific | 200 m | (Francis et al., 2005) |
|  | **6** | 0 | Central Pacific | 4000 m | (Hallam et al., 2006b) |
|  | **1** | 0 | Central Pacific | 500 m | (Hallam et al., 2006b) |
|  | 0 | **1** | Central Pacific | 130 m | (Hallam et al., 2006b) |
|  | **10** | 0 | Monterey Bay and North Pacific subtropical gyre | 4000 m | (Mincer et al., 2007) |
|  | **2** | 0 | Monterey Bay and North Pacific subtropical gyre | 500 m | (Mincer et al., 2007) |
|  | 0 | **2** | Monterey Bay and North Pacific subtropical gyre | 200 m | (Mincer et al., 2007) |
|  | 0 | **1** | Monterey Bay and North Pacific subtropical gyre | 130 m | (Mincer et al., 2007) |
|  | 2 | **33** | Monterey Bay | 30-40 m | (Francis et al., 2005) |
|  | 4 | **15** | Gulf of California | 450 m | (Beman et al., 2008) |
|  | 0 | **46** | Gulf of California | 60 m | (Beman et al., 2008) |
|  | 12 | **22** | Eastern South Pacific | Oxygen minimum zones | Molina et al., unpublished |
|  | 2 | **23** | Peruvian oxygen minimum zone | Oxygen minimum zones | (Lam et al., 2009) |
|  | **4** | 0 | North Atlantic 1.00 N 20.60 W | 2502 m | (Agogué et al., 2008) |
|  | 19 | **229** | South Atlantic (22.99S 13.36E/14.04E, coastal) | ? | Moraru et al., unpublished |
|  | 5 | **10** | Gulf of Mexico (seawater) | ? | Ye et al., unpublished |
|  | 0 | **3** | water in a coastal location of the Gulf of Mexico | ? | Liu et al., unpublished |
|  | **12** | 1 | South China Sea | 2000 m | Hu and Jiao, unpublished |
|  | **4** | 1 | South China Sea | 500 m | Hu and Jiao, unpublished |
|  | 0 | **36** | South China Sea | 100 m | Hu and Jiao, unpublished |
|  | 0 | **18** | South China Sea | 75 m | Hu and Jiao, unpublished |
|  | 5 | **12** | Deep-Ocean of the Northeastern Japan Sea (water column) | deep ocean | (Nakagawa et al., 2007) |
|  | 0 | **53** | central gyre of the Black Sea | ? | (Francis et al., 2005) |
|  | 1 | **6** | Black Sea | 100-130 m | (Lam et al., 2009) |
|  | 0 | **4** | Black Sea | ? | (Lam et al., 2009) |
|  | **1** | 0 | Tyrrhenian Sea 39.53 N 13.37 E | 3000 m | (Yakimov et al., 2009) |
|  | 0 | **2** | Deep hypersaline anoxic lake (L'Atalante, Med Sea) | 3400 M | (Yakimov et al., 2007) |
| **Hydrothermal vents** | **3** | 0 | Microbial mats at deep-sea hydrothermal fields of the Southern Mariana Trough | ? | (Kato et al., 2009) |
|  | 4 | **51** | hydrothermal vent chimneys of the Juan de Fuca ridge | >2000 m | (Wang et al., 2009) |
|  | 1 | **8** | Pacific Ocean: southwest, Lau Basin (hydrothermal vents sediments) | 2000-3000 | Dong and Shao, unpublished |
|  | 1 | **4** | methane seep surface sediments of the Okhotsk Sea | ? | Dang, unpublished |
|  | 0 | **1** | deep-sea sediments of the west Pacific continental margin | ? | (Wang et al., 2009) |
| **Sediments** | 0 | **4** | Deep-Ocean of the Northeastern Japan Sea (sediments) |  | (Nakagawa et al., 2007) |
|  | 0 | **32** | marine sediments |  | Rhee, unpublished |
|  | 0 | **75** | marine sediments |  | Park and Rhee, unpublished |
|  | 0 | **1** | coastal marine sediment |  | Yin and Zhang, unpublished |
|  | 0 | **5** | coastal sediments |  | (Santoro et al., 2008) |
|  | 0 | **5** | San Francisco Bay, Central Bay station 20, sediment |  | (Francis et al., 2005) |
|  | 0 | **3** | sediment from South San Francisco Bay |  | (Park et al., 2006) |
|  | 0 | **2** | Elkhorn Slough, CA, Vierra Marsh, sediment |  | (Francis et al., 2005) |
|  | 0 | **4** | Elkhorn Slough, CA, Hummingbird Island sediment |  | (Francis et al., 2005) |
|  | 0 | **188** | estuarine sediment | ? | (Bernhard et al., 2010) |

Table S5. Cont.

|  | **Number of clones** | **Number of clones** | **Environment** | **Depth** | **Reference** |
| --- | --- | --- | --- | --- | --- |
|  | **LAC-amoA** | **HAC-amoA** |  |  |  |
| **Sediments** | 0 | **138** | estuary sediments San Francisco bay |  | (Mosier and Francis, 2008) |
|  | 0 | **19** | estuarine sediments |  | Bano et al., unpublished |
|  | 0 | **1** | Westerschelde estuary sediment |  | (Sahan and Muyzer, 2008) |
|  | 0 | **21** | tropical marine estuary sediment |  | Singh et al., unpublished |
|  | 0 | **1** | sediments of a hypernutrified subtropical estuary: Bahia del Tobari, Mexico |  | (Beman and Francis, 2006) |
|  | 0 | **16** | salt marsh sediment |  | (Moin et al., 2009) |
|  | 0 | **28** | sand of an eelgrass zone/sediment |  | (Ando et al., 2009) |
|  | 0 | **1** | nitrifying enrichment from coastal sand of an eelgrass zone (C. Nitrosopumilus sp.) |  | Nakagawa, unpublished |
|  | 0 | **2** | tidal flat |  | Park et al., unpublished |
|  | 0 | **1** | intertidal sandy flat Douro estuary |  | Magalhaes et al., unpublished |
|  | 0 | **4** | sediments in the Changjiang Estuary |  | (Dang et al., 2008) |
| **Freshwater sediments** | 0 | **2** | sediments of Lake Taihu |  | Xiang et al., unpublished |
|  | 0 | **1** | sediments of the large subtropical shallow eutrophic Taihu Lake, China |  | Wu et al., unpublished |
|  | 0 | **1** | sediment of Lake Taihu |  | (Ye et al., 2009) |
|  | 0 | **5** | sediments of Qinghai Lake |  | Jiang et al., unpublished |
|  | 0 | **17** | roots of Myriophyllum alterniflorum, Lake Almind (freshwater sediments) |  | (Herrmann et al., 2009) |
|  | 0 | **2** | rhizosphere of Littorella uniflora, Lake Hampen |  | (Herrmann et al., 2008) |
| **Soil** | 0 | **3** | soil of a Japanese paddy field |  | Fujii et al. (In press) |
|  | 0 | **12** | temperate forest soil Japan |  | (Onodera et al., 2010) |
| **Hot springs** | 0 | **58** | Kamchatka hot springs |  | Zhao et al., unpublished |
|  | 0 | **23** | terrestrial hot springs of Iceland and Kamchatka |  | (Reigstad et al., 2008) |
|  | 0 | **3** | Mammoth Hot Spring sediment |  | (de la Torre et al., 2008) |
|  | 0 | **33** | terrestrial hot springs |  | (Zhang et al., 2008) |
|  | 0 | **1** | hot spring Nevada |  | Ye et al., unpublished |
|  | 0 | **3** | hot spring microbial mat, China: Yunnan Province |  | Jiang, unpublished |
|  | 0 | **4** | subsurface radioactive thermal spring in the Austrian Central Alps |  | (Weidler et al., 2008) |
|  | 0 | **63** | Speleothem Formation in a Geothermal Mine Adit |  | (Spear et al., 2007) |
| **Freshwater systems** | 0 | **3** | freshwater flow channel |  | Herrmann, unpublished |
|  | 0 | **1** | Tibetan lakes |  | Hu and Liu, unpublished |
|  | 0 | **3** | water in the lower Mississippi River New Orleans |  | Liu et al., unpublished |
|  | 0 | **1** | confluence of the Ohio and Mississippi River water |  | Liu et al., unpublished |
|  | 0 | **1** | freshwater river used as a drinking water source |  | Liu et al., unpublished |
|  | 0 | **3** | water of Qinghai Lake |  | Jiang et al., unpublished |
|  | 0 | **2** | Qinghai lake water |  | Jiang et al., unpublished |
|  | 0 | **89** | groundwater |  | Reed et al., unpublished |
|  | 0 | **2** | meromictic High Arctic lake |  | (Pouliot et al., 2009) |
|  | 0 | **1** | Nitrosoarchaeum limnia |  | (Blainey et al., 2011) |
| **Wastewater** | 0 | **9** | wastewater treatment plant operated with low dissolved oxygen levels and long retention times |  | (Park et al., 2006) |
| **and other environments** | 0 | **1** | enriched nitrifying activated sludge |  | Sonthiphand, unpublished |
|  | 0 | **1** | Stanley wastewater treatment plant |  | (Zhang et al., 2009) |
|  | 0 | **1** | nitrogen removal bioreactor |  | (Zhang et al., 2009) |
|  | 0 | **1** | rockwool deodorization biofilter |  | Yasuda et al., unpublished |
|  | 0 | **1** | tropical seawater tank substratum (Nitrosopumilus maritimus) |  | (Könneke et al., 2005) |
|  | 0 | **95** | aquarium biofilter |  | (Urakawa et al., 2008) |
|  | 0 | **5** | marine aquaculture biofilm |  | (Foesel et al., 2008) |
|  | 0 | **1** | Nitrosopumilus maritimus complete genome |  | (Könneke et al., 2005) |
| **Host systems** | 0 | **67** | Brazil: Guanabara Bay, Sponges |  | Turque., unpublished |
|  | 0 | **9** | marine sponges |  | (Steger et al., 2008) |
|  | 0 | **122** | Red Sea/host coral |  | Siboni et al., unpublished |
|  | 0 | **2** | coral colony |  | (Beman et al., 2007) |
|  | 0 | **10** | tissue of a colonial ascidian |  | (Martinez-Garcia et al., 2008) |
|  | 0 | **1** | Cenarchaeum symbiosum |  | (Hallam et al., 2006a) |

Agogué H, Brink M, Dinasquet J, Herndl GJ (2008). Major gradients in putatively nitrifying and non-nitrifying Archaea in the deep North Atlantic. *Nature* **456:** 788-791.

Ando Y, Nakagawa T, Takahashi R, Yoshihara K, Tokuyama T (2009). Seasonal changes in abundance of ammonia-oxidizing Archaea and ammonia-oxidizing Bacteria and their nitrification in sand of an eelgrass zone. *Microbes Environ*.

Beman JM, Francis CA (2006). Diversity of ammonia-oxidizing Archaea and Bacteria in the sediments of a hypernutrified subtropical estuary: Bahia del Tobari, Mexico. *Appl Environ Microbiol* **72:** 7767-7777.

Beman JM, Roberts KJ, Wegley L, Rohwer F, Francis CA (2007). Distribution and diversity of archaeal ammonia monooxygenase genes associated with corals. *Appl Environ Microbiol* **73:** 5642-5647.

Beman JM, Popp BN, Francis CA (2008). Molecular and biogeochemical evidence for ammonia oxidation by marine Crenarchaeota in the Gulf of California. *ISME J* **2:** 429-441.

Bernhard AE, Landry ZC, Blevins A, de la Torre JR, Giblin AE, Stahl DA (2010). Abundance of ammonia-oxidizing Archaea and Bacteria along an estuarine salinity gradient in relation to potential nitrification rates. *Appl Environ Microbiol* **76:** 1285-1289.

Blainey PC, Mosier AC, Potanina A, Francis CA, Quake SR (2011). Genome of a low-salinity ammonia-oxidizing archaeon determined by single-cell and metagenomic analysis. *Plos One* **6**.

Dang HY, Zhang XX, Sun J, Li TG, Zhang ZN, Yang GP (2008). Diversity and spatial distribution of sediment ammonia-oxidizing Crenarchaeota in response to estuarine and environmental gradients in the Changjiang Estuary and East China Sea. *Microbiology-SGM* **154:** 2084-2095.

de la Torre JR, Walker CB, Ingalls AE, Konneke M, Stahl DA (2008). Cultivation of a thermophilic ammonia oxidizing archaeon synthesizing crenarchaeol. *Environ Microbiol* **10:** 810-818.

Foesel BU, Gieseke A, Schwermer C, Stief P, Koch L, Cytryn E *et al* (2008). Nitrosomonas Nm143-like ammonia oxidizers and Nitrospira marina-like nitrite oxidizers dominate the nitrifier community in a marine aquaculture biofilm. *FEMS Microbiol Ecol* **63:** 192-204.

Francis CA, Roberts KJ, Beman JM, Santoro AE, Oakley BB (2005). Ubiquity and diversity of ammonia-oxidizing Archaea in water columns and sediments of the ocean. *Proc Natl Acad Sci USA* **102:** 14683-14688.

Hallam SJ, Konstantinidis KT, Putnam N, Schleper C, Watanabe Y, Sugahara J *et al* (2006a). Genomic analysis of the uncultivated marine Crenarchaeote Cenarchaeum symbiosum. *Proc Natl Acad Sci USA* **103:** 18296-18301.

Hallam SJ, Mincer TJ, Schleper C, Preston CM, Roberts K, Richardson PM *et al* (2006b). Pathways of carbon assimilation and ammonia oxidation suggested by environmental genomic analyses of marine Crenarchaeota. *Plos Biol* **4:** 520-536.

Herrmann M, Saunders AM, Schramm A (2008). Archaea dominate the ammonia-oxidizing community in the rhizosphere of the freshwater macrophyte Littorella uniflora. *Appl Environ Microbiol* **74:** 3279-3283.

Herrmann M, Saunders AM, Schramm A (2009). Effect of lake trophic status and rooted macrophytes on community composition and abundance of ammonia-oxidizing prokaryotes in freshwater sediments. *Appl Environ Microbiol* **75:** 3127-3136.

Kalanetra KM, Bano N, Hollibaugh JT (2009). Ammonia-oxidizing Archaea in the Arctic Ocean and Antarctic coastal waters. *Environ Microbiol* **11:** 2434-2445.

Kato S, Kobayashi C, Kakegawa T, Yamagishi A (2009). Microbial communities in iron-silica-rich microbial mats at deep-sea hydrothermal fields of the Southern Mariana Trough. *Environ Microbiol* **11:** 2094-2111.

Könneke M, Bernhard AE, de la Torre JR, Walker CB, Waterbury JB, Stahl DA (2005). Isolation of an autotrophic ammonia-oxidizing marine archaeon. *Nature* **437:** 543-546.

Konstantinidis KT, DeLong EF (2008). Genomic patterns of recombination, clonal divergence and environment in marine microbial populations. *ISME J* **2:** 1052-1065.

Lam P, Lavik G, Jensen MM, van de Vossenberg J, Schmid M, Woebken D *et al* (2009). Revising the nitrogen cycle in the Peruvian oxygen minimum zone. *Proc Natl Acad Sci USA* **106:** 4752-4757.

Martinez-Garcia M, Stief P, Diaz-Valdes M, Wanner G, Ramos-Espla A, Dubilier N *et al* (2008). Ammonia-oxidizing Crenarchaeota and nitrification inside the tissue of a colonial ascidian. *Environ Microbiol* **10:** 2991-3001.

Mincer TJ, Church MJ, Taylor LT, Preston C, Kar DM, DeLong EF (2007). Quantitative distribution of presumptive archaeal and bacterial nitrifiers in Monterey Bay and the North Pacific Subtropical Gyre. *Environ Microbiol* **9:** 1162-1175.

Moin NS, Nelson KA, Bush A, Bernhard AE (2009). Distribution and diversity of archaeal and bacterial ammonia oxidizers in salt marsh sediments. *Appl Environ Microbiol* **75:** 7461-7468.

Mosier AC, Francis CA (2008). Relative abundance and diversity of ammonia-oxidizing Archaea and Bacteria in the San Francisco Bay estuary. *Environ Microbiol* **10:** 3002-3016.

Nakagawa T, Mori K, Kato C, Takahashi R, Tokuyama T (2007). Distribution of cold-adapted ammonia-oxidizing microorganisms in the deep-ocean of the northeastern Japan Sea. *Microbes Environ* **22:** 365-372.

Onodera Y, Nakagawa T, Takahashi R, Tokuyama T (2010). Seasonal change in vertical distribution of ammonia-oxidizing Archaea and Bacteria and their nitrification in temperate forest soil. *Microbes Environ* **25:** 28-35.

Park HD, Wells GF, Bae H, Criddle CS, Francis CA (2006). Occurrence of ammonia-oxidizing Archaea in wastewater treatment plant bioreactors. *Appl Environ Microbiol* **72:** 5643-5647.

Pouliot J, Galand PE, Lovejoy C, Vincent WF (2009). Vertical structure of archaeal communities and the distribution of ammonia monooxygenase A gene variants in two meromictic High Arctic lakes. *Environ Microbiol* **11:** 687-699.

Reigstad LJ, Richter A, Daims H, Urich T, Schwark L, Schleper C (2008). Nitrification in terrestrial hot springs of Iceland and Kamchatka. *FEMS Microbiol Ecol* **64:** 167-174.

Sahan E, Muyzer G (2008). Diversity and spatio-temporal distribution of ammonia-oxidizing Archaea and Bacteria in sediments of the Westerschelde estuary. *FEMS Microbiol Ecol* **64:** 175-186.

Santoro AE, Francis CA, de Sieyes NR, Boehm AB (2008). Shifts in the relative abundance of ammonia-oxidizing Bacteria and Archaea across physicochemical gradients in a subterranean estuary. *Environ Microbiol* **10:** 1068-1079.

Spear JR, Barton HA, Robertson CE, Francis CA, Pace NR (2007). Microbial community biofabrics in a geothermal mine adit. *Appl Environ Microbiol* **73:** 6172-6180.

Steger D, Ettinger-Epstein P, Whalan S, Hentschel U, de Nys R, Wagner M *et al* (2008). Diversity and mode of transmission of ammonia-oxidizing archaea in marine sponges. *Environ Microbiol* **10:** 1087-1094.

Urakawa H, Tajima Y, Numata Y, Tsuneda S (2008). Low temperature decreases the phylogenetic diversity of ammonia-oxidizing archaea and bacteria in aquarium biofiltration systems. *Appl Environ Microbiol* **74:** 894-900.

Wang SF, Xiao X, Jiang LJ, Peng XT, Zhou HY, Meng J *et al* (2009). Diversity and abundance of ammonia-oxidizing Archaea in hydrothermal vent chimneys of the Juan de Fuca Ridge. *Appl Environ Microbiol* **75:** 4216-4220.

Weidler GW, Gerbl FW, Stan-Lotter H (2008). Crenarchaeota and their role in the nitrogen cycle in a subsurface radioactive thermal spring in the Austrian central Alps. *Appl Environ Microbiol* **74:** 5934-5942.

Yakimov MM, La Cono V, Denaro R, D'Auria G, Decembrini F, Timmis KN *et al* (2007). Primary producing prokaryotic communities of brine, interface and seawater above the halocline of deep anoxic lake L'Atalante, Eastern Mediterranean Sea. *ISME J* **1:** 743-755.

Yakimov MM, La Cono V, Denaro R (2009). A first insight into the occurrence and expression of functional amoA and accA genes of autotrophic and ammonia-oxidizing bathypelagic Crenarchaeota of Tyrrhenian Sea. *Deep-Sea Res Part II* **56:** 748-754.

Ye WJ, Liu XL, Lin SQ, Tan J, Pan JL, Li DT *et al* (2009). The vertical distribution of bacterial and archaeal communities in the water and sediment of Lake Taihu. *FEMS Microbiol Ecol* **70:** 263-276.

Zhang CL, Ye Q, Huang ZY, Li WJ, Chen JQ, Song ZQ *et al* (2008). Global occurrence of archaeal amoA genes in terrestrial hot springs. *Appl Environ Microbiol* **74:** 6417-6426.

Zhang T, Jin T, Yan Q, Shao M, Wells G, Criddle C *et al* (2009). Occurrence of ammonia-oxidizing Archaea in activated sludges of a laboratory scale reactor and two wastewater treatment plants. *J Appl Microbiol* **107:** 970-977.
